# ASPHD1 Is a tumor-suppressive and prognostic marker in glioma

**DOI:** 10.3389/fonc.2025.1694116

**Published:** 2026-01-02

**Authors:** Jinhua Yang, Fenfei Gao, Xiaowan Wang, Yanmei Zhang, Shuangtao Li

**Affiliations:** 1 Department of Pharmacology, Shantou University Medical College, Shantou, China; 2 Department of Neurobiological Science, Shantou University Medical College, Shantou, China

**Keywords:** ASPHD1, glioma, proliferation, neuronal differentiation, calcium signaling, diagnostic biomarker, prognostic biomarker

## Abstract

**Introduction:**

Glioma is a highly aggressive primary brain tumor. To identify novel prognostic biomolecules and potential therapeutic targets, we investigated Aspartate betahydroxylase domain-containing protein 1 (ASPHD1), encoded by the *ASPHD1* gene and predicted to function as a Fe (II)/2-oxoglutarate–dependent dioxygenase involved in peptide amino-acid modification. ASPHD1 has not previously been characterized in glioma.

**Methods:**

We assessed its expression and prognostic value using transcriptomic data from the Cancer Genome Atlas (TCGA), Gene Expression Omnibus (GEO), and Chinese Glioma Genome Atlas (CGGA). Co-expressed gene enrichment and DNA-methylation analyses were performed, and associations between ASPHD1 expression and overall survival (OS) were evaluated. Gene Ontology and KEGG analyses of ASPHD1 coexpressed genes indicated enrichment in synaptic signaling, ion-channel activity, and calcium-signaling pathways, suggesting a link with neuronal and synaptic function.

**Results:**

ASPHD1 expression showed a pronounced inverse correlation with WHO grade, and higher ASPHD1 expression was associated with prolonged OS; multivariable Cox models identified low ASPHD1 as an independent adverse prognostic factor. Pan-cancer analysis further revealed that higher ASPHD1 expression was associated with longer OS in skin cutaneous melanoma (SKCM), uveal melanoma (UVM), and mesothelioma (MESO). In glioma cell lines, ASPHD1 overexpression suppressed proliferation, migration, and invasion in vitro, and inhibited tumor growth in a subcutaneous U87 xenograft model. ASPHD1 overexpression also upregulated neuronal differentiation–related genes, produced more negative resting membrane potentials on whole-cell patch-clamp recordings, enhanced depolarization-evoked Ca^2+^ transients on calcium imaging, and increased NeuN protein expression.

**Discussion:**

Together, these findings identify ASPHD1 as a favorable prognostic biomarker in glioma and suggest that high ASPHD1 expression restrains glioma progression while promoting neuron-like differentiation of glioma cells.

## Introduction

Glioma stands out as the most common and formidable type of primary brain tumors found in adults (1). Typical and common molecular alterations in glioma include isocitrate dehydrogenase (IDH) mutations, 1p/19q codeletion, TP53 mutations, Phosphatase and Tensin Homolog(PTEN) loss, epidermal growth factor receptor (EGFR) amplification and O6-methylguanine DNA methyltransferase (MGMT) promoter methylation. Specific mutations in the genes responsible for IDH 1/2 appear to define tumors linked to a younger age and a more favorable prognosis. 1p/19q codeletion has been repeatedly shown to be a favorable prognostic factor (2). Mutations in the TP53 tumor suppressor gene are frequently observed in glioma, particularly in astrocytoma and glioblastoma, and TP53 mutations can be used as a distinguishing feature to distinguish between glioma and gliosis (3). PTEN deficiency is a common alteration in glioblastoma, IDH-wildtype (CNS WHO grade 4). PTEN deletion in gliomas activates YAP1, which upregulates LOX expression, leading to macrophage recruitment to the tumor microenvironment through the β1 integrin-PYK2 pathway, enhancing glioma survival and angiogenesis. Therefore, PTEN loss not only promotes tumor cell survival but also influences tumor progression by modulating immune cells within the tumor microenvironment (4). Alterations in the EGFR are present in approximately 57% of glioblastoma cases, indicating a significant molecular profile shift in these tumors (5). The wild-type form of EGFR stimulates the STAT3 pathway, thereby promoting oncogenic transformation (6, 7). In IDH-mutant gliomas, methylation within the promoter region of MGMT serves as a prognostic biomarker for extended disease-free survival and life expectancy, offering valuable prognostic information (8). However, the prognostication of gliomas, especially glioblastoma, remains poor. With the advancement of medicine, new targets for glioma treatment are emerging, such as VEGF (vascular endothelial growth factor) (9), HDACs (10, 11). Present advancements are centered on targeting the molecular traits that propel the malignant phenotype, altered signal transduction such as TGF-β signaling pathway (12) and angiogenesis.

We search for new possible targets at glioma. ASPHD1 is predicted to have dioxygenase activity and be involved in peptidyl-amino acid modification, as well as be an integral membrane component. The *ASPHD1* gene is linked to conditions such as spondylocostal dysostosis, schizophrenia, and the syndrome characterized by the deletion near the proximal end of chromosome 16’s short arm. According to functional classification systems related to genomics, ASPHD1 is implicated in activities involving oxygen-dependent enzymes. [provided by Alliance of Genome Resources, Apr 2022].

In the past two years, there has been a gradual increase in literature reports on the *ASPHD1* gene, predominantly based on bioinformatics analyses, with fewer experimental studies. Sun et al., through analysis of multiple public databases, found that patients with melanoma who have higher expression of ASPHD1 have a longer overall survival compared to those with lower expression. ASPHD1 may be involved in cancer progression through its control of alpha-ketoglutarate-dependent dioxygenases (13). Asadnia and colleagues, using machine learning integrated with bioinformatics methods, identified the *ASPHD1* gene as a potential prognostic marker for early-stage colorectal cancer, valuable for prognosis assessment. They also determined that the rs925939730 variant in ASPHD1 might be involved in the regulation of gene expression (14). Cao et al. discovered that hsa_circ_0006847 (a circular RNA from the *ASPHD1* gene) is expressed at reduced levels in gastric cancer tissues and cells, and experimental evidence suggests that this circRNA facilitates the interaction between EIF4A3 and SYNPO2, potentially offering a new target for gastric cancer treatment (15).

However, there is currently no relevant research on ASPHD1 in glioma. In our study, we investigated the levels of ASPHD1 in glioma patients by utilizing data from the TCGA, CGGA, and GEO databases. By performing a comprehensive analysis, we evaluate the genomic interaction networks associated with ASPHD1 in glioma and further explore its function in intratumor communication. Our findings suggest that ASPHD1 may serve as a promising new candidate for the diagnosis and management of glioma.

## Materials and methods

### Data collection

Information for ASPHD1 expression in most of the tumors can be found on the GEPIA website. We procured gene expression profiles, DNA methylation patterns, and patient clinical data for TCGA GBMLGG from the TCGA repository. Moreover, we sourced CGGA datasets from the specified CGGA link. The glioma GEO dataset GSE7696 was acquired from the NCBI’s resource. Our investigation involved glioma cases from the TCGA, CGGA, and GEO repositories, specifically those with available transcriptional profiles and clinical outcome data. The threshold for the ASPHD1 category was established by adopting the median of the mRNA expression rankings in the glioma collection. This classification enabled us to group samples into categories with either diminished ASPHD1 expression or augmented ASPHD1 expression. In addition, we executed a methodical analysis of ASPHD1 protein levels across varying cancer stages, employing immunohistochemical imagery obtained from the Human Protein Atlas (16).

### Analyzing diagnostic and prognostic value

To assess whether ASPHD1 adds prognostic information beyond established markers, we built Cox proportional hazards models for overall survival including age and IDH mutation status with or without ASPHD1 expression in the CGGA mRNAseq_325 (training) and CGGA mRNAseq_693 (validation) cohorts. For each model, time-dependent ROC curves and AUCs at 3- and 5-year OS were calculated. Risk scores from the combined model were used to stratify patients into quartiles. Model calibration was evaluated by plotting predicted versus observed 3-year mortality. Clinical utility and net benefit were assessed using decision-curve analysis (DCA) at 3-year OS, comparing age alone, age + IDH, and age + IDH + ASPHD1. We stratified the patients into two groups characterized by above-median and below-median ASPHD1 levels. Survival curves were constructed using R’s survival analysis package. The primary measure of interest was the duration of overall survival. Analyses were performed using the Cox proportional hazards model to assess the impact of various prognostic factors on patient survival outcomes.

### Analysis of gene functional enrichment

We pinpointed genes exhibiting either positive or negative associations with *ASPHD1* by using the LinkedOmics platform. We selected the 50 genes with the strongest positive correlation and the 50 genes with the strongest negative correlation with *ASPHD1* to construct heatmaps. We conducted Gene Ontology (GO) function annotation analysis using the GO database and Kyoto Encyclopedia of Genes and Genomes (KEGG) pathway annotation analysis using the KEGG database. The enrichment analyses for both GO and KEGG were carried out utilizing R software.

### Analysis of the PPI network and modules

We established a protein-protein interaction (PPI) network at a confidence threshold exceeding 0.6 utilizing the STRING database, version 11.5, encompasses data on over 14,000 species, upwards of 60 million proteins, and in excess of 20 billion interactions. These interactions encompass both direct physical contacts and indirect functional associations between proteins. The STRING database facilitates the retrieval of established protein interactions, aiding in the elucidation of complex regulatory networks within organisms. We utilized Network visualization tool (Cytoscape, version 3.9.0) to construct PPI interactions networks.

### Analysis pertaining to methylation

We retrieved DNA methylation datasets from the TCGA database, to assess the relationship between methylation density and gene expression, we employed Spearman’s correlation analysis.

### Pan cancer study

The association of ASPHD1 with pan-cancer expression and survival was determined employing the web-based tool GEPIA for Gene Expression Profiling Interactive Analysis (17). TISIDB was engaged to assess how ASPHD1 levels correlate with overall patient survival in diverse forms of cancer. For this extensive malignancy evaluation, we constructed survival outcome curves, namely Kaplan-Meier plots, utilizing the patient data ensemble from TCGA.

### Tumor immunology analysis

CIBERSORT, short for Cell-type Identification By Estimating Relative Subsets Of RNA Transcripts, was utilized to calculate the cellular make-up of heterogeneous tissue samples from tumor gene expression data (18). ESTIMATE, an acronym for Estimation of Stromal and Immune cells in Malignant Tumor tissues using Expression data, is a methodology that uses gene expression patterns to gauge the stromal and immune cell percentages within tumor samples. This approach was applied to determine the extent of immune cell presence (immune score), the stromal proportion (stromal score), a combined stromal-immune metric (ESTIMATE score), and the level of tumor purity for each glioma sample analyzed.

### Cultivation and growth analysis of cells

The U87 and U251 human glioblastoma cell lines were obtained from Professor Jie Wu (Neuroscience Department, Shantou University Medical College, Shantou, China) and were authenticated by short tandem repeat (STR) profiling before use. The identity of the cell lines was confirmed by morphology and growth characteristics, and all cell lines were routinely tested for mycoplasma contamination using a PCR-based assay, with only mycoplasma-negative cells used for experiments. U251 and U87 were cultured in Dulbecco’s Modified Eagle Medium (DMEM) supplied by Invitrogen, supplemented with 10% Fetal Bovine Serum (FBS), and kept at a stable 37 °C in a humidified atmosphere of 5% CO2. To assess cell survival and proliferation, the CCK-8 assay was conducted according to the protocol recommended by Dojindo (Japan). This assay is crucial for determining active cell numbers in the culture. U87 and U251 cell populations, at a concentration of 3,000 units per designated compartment, were seeded into 96-well plates and then nurtured over intervals spanning one to four days. Post incubation, a 10 μL aliquot of CCK-8 reagent was applied to every well, followed by a further 2-hour incubation at 37 °C. Subsequently, the optical density was then determined at a 450 nm wavelength with a microplate spectrophotometer from Bio-Rad.

### Clonogenic assay

Cells were trypsinized, counted using a hemocytometer, and seeded into 6-well plates at a density of 200 cells per well. Cells were incubated for 14 days to allow colony formation. During this period, the medium was changed every 3–4 days. After the incubation period, cells were gently washed with PBS (phosphate-buffered saline). Colonies were fixed with 4% paraformaldehyde for 15 minutes at room temperature. Fixed colonies were then stained with 0.5% crystal violet solution for 30 minutes. Stained colonies were washed with water and allowed to air-dry. The number of colonies and the size of colonies provide insight into the proliferative capability of the cells after treatment.

### 5-ethynyl-2′-deoxyuridine incorporation assay

Cell proliferation was evaluated using a 5-ethynyl-2′-deoxyuridine (EdU) incorporation assay. Cultured cells were incubated with 10 μM EdU working solution at 37°C for 2 h. EdU labeling was performed with the BeyoClick EdU-594 Cell Proliferation Assay Kit (Beyotime, C0078S) according to the manufacturer’s instructions. For flow-cytometric analysis, cells were collected, fixed, permeabilized, and subjected to the click reaction in suspension; EdU-positive cells were quantified on a Celula Sparrow 2040 flow cytometer, and data were analyzed using AISFCM software (AISFCM-analyze, Celula, V1.0.0.180522). In parallel, cells grown in culture dishes were fixed after EdU incubation, processed with the same click reaction, counterstained with DAPI, and imaged by fluorescence microscopy to visualize EdU-positive nuclei. All experiments were independently repeated three times.

### Generation of stable ASPHD1-overexpressing and ASPHD1-knockdown glioma cell lines

The YOE-LV001-ASPHD1 lentiviral vector (CMV–ASPHD1–T2A–Puro) and the corresponding empty control vector YOE-LV001-Ctrl were purchased from Guangzhou Ubigene Biosciences Co., Ltd. (Guangzhou, China) (**Supplementary Figure S1A**). U87 and U251 glioma cells were seeded in 6-well plates at a density of 2–3 × 10^5 cells per well, allowed to adhere overnight, and then infected with YOE-LV001-ASPHD1 or YOE-LV001-Ctrl lentivirus at a multiplicity of infection (MOI) of 10 in complete medium containing 8 μg/mL polybrene. After 24 h, the viral supernatant was replaced with fresh complete medium and cells were cultured for an additional 24–48 h, followed by selection with 2 μg/mL puromycin (Beyotime, China) for 5–7 days until all cells in non-infected control wells were eliminated. Puromycin-resistant cells were subjected to single-cell cloning by limiting dilution into 96-well plates, and wells containing a single colony were expanded as individual clones. ASPHD1 expression in each clone was examined by quantitative real-time PCR (qRT-PCR) and Western blotting, and several independent clones with robust ASPHD1 overexpression were pooled in approximately equal proportions to establish stable ASPHD1-overexpressing U87 and U251 cell lines; vector-control lines were generated in parallel using the empty vector. For loss-of-function studies, ASPHD1 knockdown was achieved using a lentiviral shRNA approach: a short hairpin RNA targeting human ASPHD1 (shASPHD1, 5′-GATAACCAGATGCAACGTGAA-3′) and a non-targeting scramble shRNA control (shNC, 5′-CCTAAGGTTAAGTCGCCCTCG-3′) were cloned into a puromycin-selectable lentiviral vector under the control of the U6 promoter, packaged into lentiviral particles, and used to infect U251 cells in the presence of 8 μg/mL polybrene. Infected cells were selected with 2 μg/mL puromycin for 5–7 days and expanded as pooled stable shASPHD1 and shNC populations. All stable lines were maintained in DMEM supplemented with 10% FBS and 1 μg/mL puromycin, passaged at a ratio of 1:3–1:5 every 2–3 days, and used for experiments within 10 passages after establishment. Overexpression and knockdown efficiency were confirmed at both the mRNA and protein levels by qRT-PCR and Western blotting.

### Isolation of RNA and quantitative RT-PCR analysis

RNA isolation from the cell cultures was performed using the RNA-easy Isolation Kit (Vazyme), according to the protocol provided. Subsequently, the extracted RNA was transcribed into cDNA utilizing the PrimeScript RT Reagent Kit with gDNA Eraser (TaKaRa, RR047A) to prepare for PCR amplification. The qPCR analysis was executed using the 7500 Real-Time PCR System from Applied Biosystems, which was equipped with the PrimeScript RT Reagent Kit containing gDNA Eraser for enhanced precision (TaKaRa, RR047A). The thermal cycling settings included an initial step at 95°C for half a minute, succeeded by 40 cycles at 95°C for 5 seconds and 60°C for 34 seconds. GAPDH mRNA expression served as the baseline to normalize mRNA levels, employing the comparative 2^-ΔΔCT technique for quantification. The primer sequences used for qRT-PCR are listed in **Table 1**.

**Table 1:** Primer sequences.

Primer name	Forward primer (5′-3′)	Reverse primer (5′-3′)
ASPHD1	GGATGGGTAGAGTGAAGGCG	AGGCAGGTCGTGGTAGAAAAG
Nestin	CTGCTACCCTTGAGACACCTG	GGGCTCTGATCTCTGCATCTAC
SOX2	CACATGAACGGCTGGAGCAA	GGAGTGGGAGGAAGAGGTAAC
GFAP	ACATCGAGATCGCCACCTAC	ACATCACATCCTTGTGCTCC
S100β	TGGCCCTCATCGACGTTTTC	ATGTTCAAAGAACTCGTGGCA
Galc	TATTTCCGAGGATACGAGTGGT	CCAGTCGAAACCTTTTCCCAG
OLIG2	CCAGAGCCCGATGACCTTTTT	CACTGCCTCCTAGCTTGTCC
TUBB3	CCAAGCGGCTACACGTCTC	CGTCCCATTCAGCTTCTCCC
MAP2	GGCCAAGGGTCACTACACG	GCAGTCGCAGTTTTCACACTC
RBFOX3	CCAAGCGGCTACACGTCTC	CGTCCCATTCAGCTTCTCCC
SYP	CTCGGCTTTGTGAAGGTGCT	CTGAGGTCACTCTCGGTCTTG
GAPDH	GGTCGGAGTCAACGGATTTGGTC	CCTCCGACGCCTGCTTCACCAC

### Western blotting

Glioma cells underwent lysis with a Membrane and Cytoplasmic Protein Extraction Kit (Sangon Biotech), after which we quantified the protein content using the Bradford Protein Assay kit (Beyotime, China). A quantity of 50 μg of the protein was then subjected to electrophoresis on a 12% SDS-PAGE gel. The resulting nitrocellulose sheet was treated with 5% skim milk to block non-specific binding for 60 minutes. The sheet was then incubated with primary antibodies against ASPHD1 (1:1000, sourced from Abcam, ab197301), NeuN rabbit polyclonal antibody (1:1000, provided by CST, #24307), Histone H3 rabbit polyclonal antibody (1:3000, provided by CST, ABL1141) and anti-Na^+^-K^+^-ATPase α1 rabbit polyclonal antibody (1:1000, provided by Abbkine, #4499). GFAP rabbit polyclonal antibody (1:3000, provided by CST, #12389), HSP90 (1:6000, provided by proteintech, SC-13119)This step was succeeded by the application of secondary antibodies from Cell Signaling Technology. For band detection, we utilized a chemiluminescence detection system (ECL; Solarbio, China).

### Cell cycle distribution

Cell cycle distribution was analyzed by flow cytometry. U87 and U251 cells transfected with ASPHD1 overexpression plasmid (ASPHD1 OE) or empty vector control were harvested after 48 hours of transfection. For each independent experiment, one well per condition from a 6-well plate was collected as one sample for both U87 and U251 cells, yielding three independent biological replicates per condition (n = 3). Cells were fixed with 70% ice-cold ethanol at 4 °C overnight. Subsequently, cells were washed with PBS and incubated with RNase A solution (100 μg/ml) for 30 min at 37°C, followed by staining with propidium iodide (PI, 50 μg/ml) in the dark at room temperature for 30 min. Stained cells were then analyzed using a flow cytometer (Celula sparrow2040, CHINA), and 2 × 10^4 events were collected for each sample. The percentages of cells in sub-G1, G0/G1, S, and G2/M phases were quantified using AISFCM software (AISFCM-analyze, Celula, V1.0.0.180522). Data are presented as mean ± SD from three independent experiments.

### Assessment of Cell migration and invasive ability

In the scratch assay, cells from various groups were detached using trypsin and seeded into 6-well plates at dense populations. Once the cells achieved high confluence (>90%), a micropipette tip was used to make a linear scratch in the monolayer of cells. Images were captured immediately (time zero) and 24 hours post-scratch. The area covered by the cell monolayer to fill the scratch during this period was quantified using ImageJ software.

Transwell assays were conducted using chamber inserts from Corning Incorporated. For the invasion assay, the inserts were coated with a Matrigel layer (Beyotime, China) to simulate extracellular matrix conditions. Transfected glioma cells were seeded into the top chambers in serum-free media to avoid serum-induced effects. For the migration assay, the chamber inserts were not coated with Matrigel. In both assays, the bottom chambers were filled with media containing 10% fetal bovine serum (FBS) to serve as a chemoattractant.

The cells were allowed to migrate or invade for 18 hours at 37°C in a humidified atmosphere of 5% CO2. Post-incubation, cells remaining on the upper surface of the membrane were gently removed using a sterile cotton applicator. Cells that migrated or invaded to the bottom surface of the membrane were fixed with methanol, stained with 0.1% crystal violet, and then air-dried. The number of cells was quantified by counting five random microscopic fields per well using a light microscope. Statistical analysis of the data was performed using a paired Student’s t-test, with p-values below 0.05 considered indicative of statistical significance.

### Whole-cell patch-clamp recordings

Resting membrane potentials were recorded using whole-cell patch-clamp in U251 cells stably expressing ASPHD1 (ASPHD1-OE) or empty vector. Cells were plated on glass coverslips 24 h before recording. Coverslips were transferred to a recording chamber and continuously perfused with an external solution containing (in mM): 140 NaCl, 5 KCl, 2 CaCl_2_, 1 MgCl_2_, 10 HEPES, 10 glucose, pH 7.4 (NaOH), ~300 mOsm. Patch pipettes (3–5 MΩ) were pulled from borosilicate glass and filled with an internal solution containing (in mM): 140 K-gluconate, 5 KCl, 2 MgCl_2_, 0.2 EGTA, 10 HEPES, 2 Mg-ATP, 0.3 Na-GTP, pH 7.3 (KOH), ~290 mOsm. Recordings were obtained at room temperature using a patch-clamp amplifier (MultiClamp 700B, Molecular Devices) and pCLAMP software. After establishing the whole-cell configuration, cells were switched to current-clamp mode with injected current set to 0 pA (I = 0). The membrane potential was allowed to stabilize for 3–5 min, and the resting membrane potential was determined as the average voltage over a 60-s period. Only cells with stable access resistance (<20 MΩ) and no obvious leak increase during recording were included in the analysis.

### Calcium imaging

Calcium imaging was performed in U251 cells using Fluo-4 AM. For gain-of-function experiments, ASPHD1-OE and vector control cells were plated on glass-bottom dishes and cultured overnight. For loss-of-function experiments, U251 shNC and shASPHD1 cells were prepared in the same way. Cells were loaded with 2 μM Fluo-4 AM (Invitrogen) in HEPES-buffered extracellular solution (in mM: 140 NaCl, 5 KCl, 2 CaCl_2_, 1 MgCl_2_, 10 HEPES, 10 glucose, pH 7.4) for 15 min at 37°C, washed several times with dye-free extracellular solution at room temperature, and then imaged on a Zeiss LSM 880 confocal microscope. Time-lapse images were acquired at 1 frame/s.

During the acquisition, cells were initially superfused with the control solution (0.1% DMSO in Ringer’s solution). Starting at 50 s, acetylcholine (ACh, 1 mM) was continuously applied using a perfusion system to evoke depolarizing stimulation. Regions of interest (ROIs) were drawn on individual cells, and the mean fluorescence intensity F(t) was extracted using ImageJ. Ca²^+^ signals were expressed as ΔF/F_0_, where F_0_ was the baseline fluorescence averaged over 30 frames before ACh perfusion. For each cell, the peak ΔF/F_0_ in response to stimulus and the number of responding cells were quantified. All imaging and analysis parameters were kept identical across experimental groups.

### Animal study

All animal experiments were conducted in accordance with the Guidelines for the Care and Use of Laboratory Animals and the Institutional Code of Ethics for Animal Experiments. For the xenograft experiments, 5-week-old female BALB/c-nu mice (purchased from Guangdong Yaokang Biotechnology Co., Ltd.) were randomly divided into two groups (n = 5 each) using a random number table and subcutaneously inoculated with 1 × 10^6^ U87 cells stably infected with lentiviruses (Vector or ASPHD1 OE) in PBS. During the observation period, one mouse in the ASPHD1 OE group died accidentally from a non–tumor-related cause and was excluded from the endpoint analysis. Thus, final tumor volume and weight analyses were performed with n = 5 mice in the Vector group and n = 4 mice in the ASPHD1 OE group. Once subcutaneous tumors became palpable, tumor length (the longest diameter) and width (the shortest diameter perpendicular to the length) were measured with a digital caliper every three days. Tumor volumes (V) were calculated using the formula V = (length × width²)/2. When reaching moribund condition or the tumor length was more than 15 mm, the mice were euthanized and their tumors were harvested for analysis, photography, and histological examination. The investigator was blinded to the group allocation of the mice during the experiment. The sample size is described in the corresponding figure legend. Mice were euthanized by brief exposure to isoflurane in a closed chamber, using an isoflurane concentration of 5–10% for rapid inhalation anesthesia. All procedures were approved by the Institutional Animal Care and Use Committee and conducted in accordance with ethical guidelines.

### Statistical methods

To analyze gene expression differences between glioma patients and normal peoples, the limma package within the R environment was utilized. After completing the analysis, we create a volcano plot using the ggplot2 package to visualize the results. For the analysis of group differences, we applied both the t-test, which assumes normal distribution and equal variances to compare group means, and the Mann-Whitney U test, a non-parametric alternative that compares median values without assuming a specific data distribution, thus providing robustness against non-normality and outliers. To investigate differences in clinical parameters between groups with low versus high expression levels of ASPHD1, the chi-square test was used, a statistical method that evaluates the significance of categorical data associations. Correlation analyses were conducted using Pearson and Spearman techniques. The significance of survival-related p-values was determined using the log-rank method. R software (version 4.2.2) alongside SPSS (version 25.0) was employed for the statistical computations. We achieved data visualization using GraphPad Prism (version 8.0) in conjunction with R. We regarded differences as statistically significant if the P value was below 0.05. For all analyses involving multiple comparisons (including functional enrichment and immune cell–related inferences), P values were adjusted for multiple testing using the Benjamini–Hochberg false discovery rate (FDR) procedure. Unless otherwise specified, terms or features with FDR-adjusted P values < 0.05 were considered statistically significant.

### Immunofluorescence staining of cryosections

The immunofluorescence analysis was performed on cryopreserved tissue sections as previously described with modifications. Briefly, fresh tissues were embedded in optimal cutting temperature (OCT) compound (Yeasen 36309ES61) and snap-frozen in liquid nitrogen. Cryosections (8 μm thickness) were prepared using a cryostat (Leica CM1950, Germany) at −20°C and mounted onto poly-L-lysine-coated slides. Sections were fixed in 4% paraformaldehyde (PFA, Sigma-Aldrich) for 15 min at room temperature (RT), permeabilized with 0.4% Triton X-100 in PBS for 10 minutes, and blocked with 5% normal goat serum for 1h at RT. Primary antibodies against ASPHD1 (1:100, sourced from Abcam, ab197301) and corresponding isotype controls were applied overnight at 4°C in a humidified chamber. After three PBS washes, sections were incubated with Cy3-labeled goat anti-rabbit secondary antibody (A0516, Beyotime) for 1 h at RT. Nuclei were stained with 2-(4-amidinophenyl)-6-indolecarbamidine dihydrochloride (DAPI, C1002, Beyotime) for 5 min. Slides were mounted with Fluoromount-G (SouthernBiotech) and imaged using a confocal microscope (Zeiss LSM 880) with ZEN imaging software. Three independent replicates were analyzed, and fluorescence intensity was quantified using ImageJ (NIH) with background subtraction.

### RNA sequencing and data analysis

Total RNA was extracted from glioma cell samples. Only high-quality RNA samples were used for downstream analysis. RNA-seq library construction and sequencing were performed by Novogene Co., Ltd. (Beijing, China). Sequencing libraries were prepared using the Illumina TruSeq RNA Sample Preparation Kit, and paired-end sequencing (2 × 150 bp) was carried out on an Illumina NovaSeq 6000 platform.

Raw sequencing reads were subjected to quality control using FastQC, and adapters and low-quality reads were removed with Trimmomatic. Clean reads were aligned to the human reference genome (GRCh38) using HISAT2. Gene expression levels were quantified using featureCounts, and differential gene expression analysis was performed using DESeq2. Genes with |log2FoldChange| ≥ 1 and adjusted p-value < 0.05 were considered significantly differentially expressed. KEGG pathway enrichment analysis was conducted using the clusterProfiler package in R, and gene set enrichment analysis (GSEA) was performed using GSEA software (Broad Institute).

## Results

### ASPHD1 is expressed at low levels in glioma tissues

**Figure 1:**
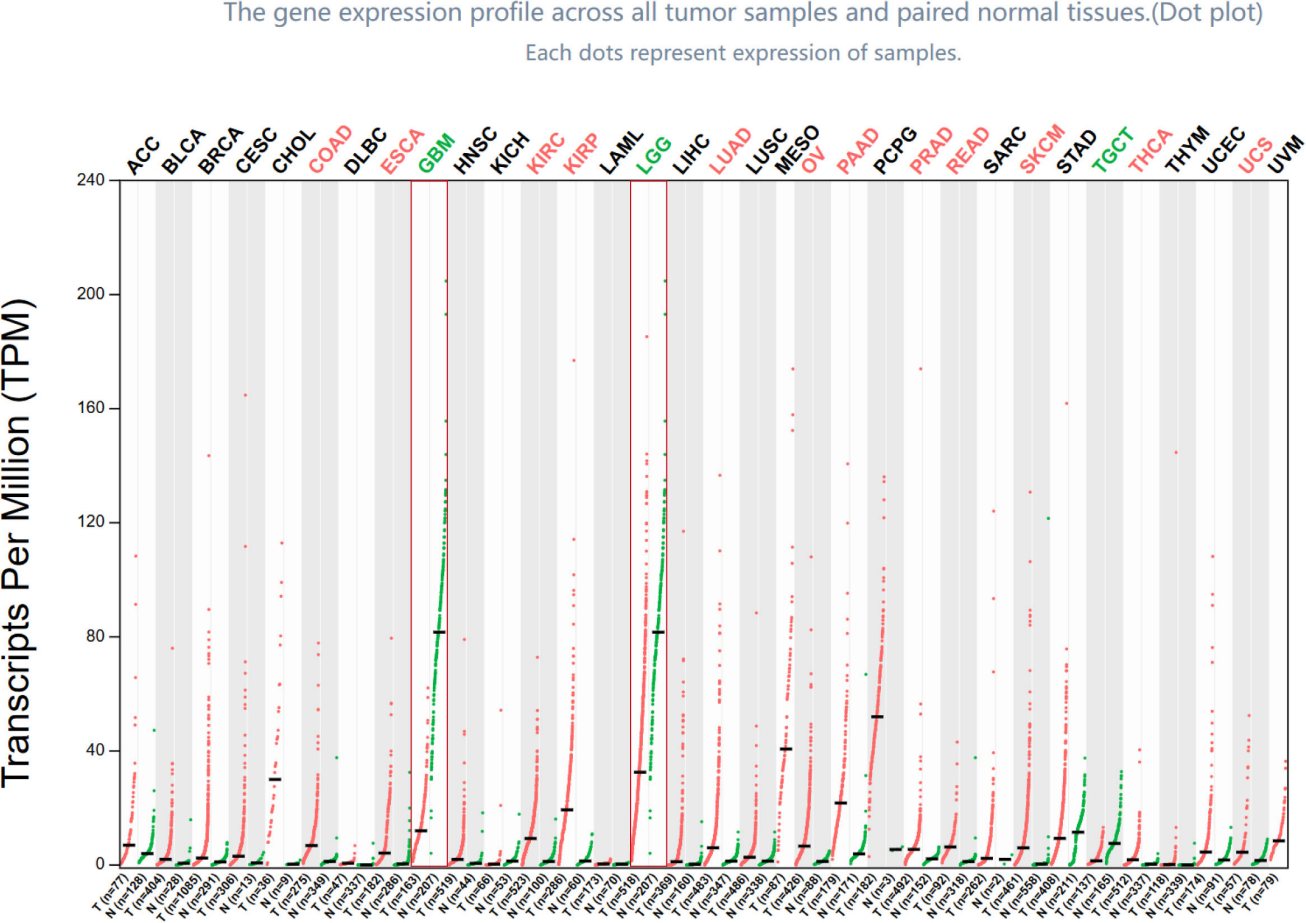
Expression levels of 36 tumors in the dot plot. The comparison of ASPHD1 mRNA level between glioma (included GBM and LGG) and normal tissues.

**Figure 2:**
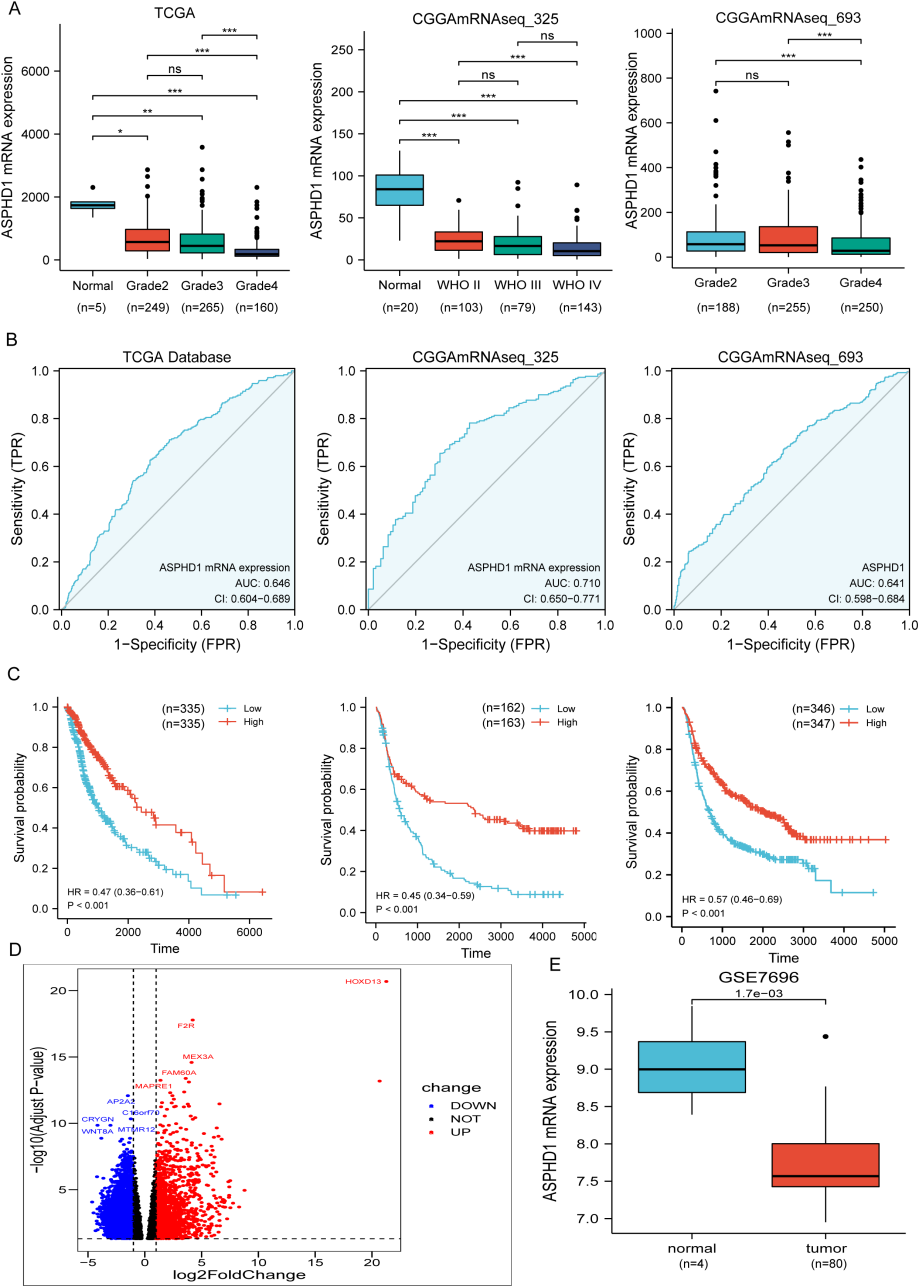
Expression and diagnostic and prognostic capabilities of ASPHD1 in glioma. **(A)** Comprehensive Analysis of ASPHD1 mRNA Expression in Glioma: Comparisons Between Normal Tissues and Across Different Grades in TCGA and CGGA Datasets. **(B)** ROC curves were generated to verify the diagnostic performance of ASPHD1 expression. **(C)** K-M survival analysis was performed to determine differences in glioma between H-ASPHD1 and L-ASPHD1 groups. **(D)** Volcano plot of DEGs between glioma and normal tissues in TCGA. Green dots represent genes that are underexpressed relative to normal tissues, while red dots represent overexpressed genes. **(E)** Comparison of ASPHD1 mRNA expression levels between glioma and normal tissues in GSE7696. ns. p > 0.05, *p < 0.05, **p < 0.01, ***p < 0.001.

ASPHD1 expression in various tumor types is shown in **Figure 1**. ASPHD1 is underexpressed in GBM, and also has a tendency to be underexpressed in lower grade glioma (LGG). The initial quantification of ASPHD1 transcriptional abundance was conducted within TCGA, consisting of a cohort of 674 glioma samples and 5 normal samples. The CCGA325 cohort consisted of 325 glioma samples and 20 normal samples, and the CCGA693 cohort was comprised of 693 glioma samples and no normal samples. Expression data from glioma cohorts obtained from the GEO database (GSE7696) was employed for Further confirm. In glioma samples compared to normal tissues, patient groups from the TCGA, GEO, and CGGA databases exhibited reduced ASPHD1 mRNA levels. Furthermore, ASPHD1 mRNA expression progressively decreased with increasing glioma grade. (**Figures 2A, E**) (*P* < 0.05).

**Figure 3:**
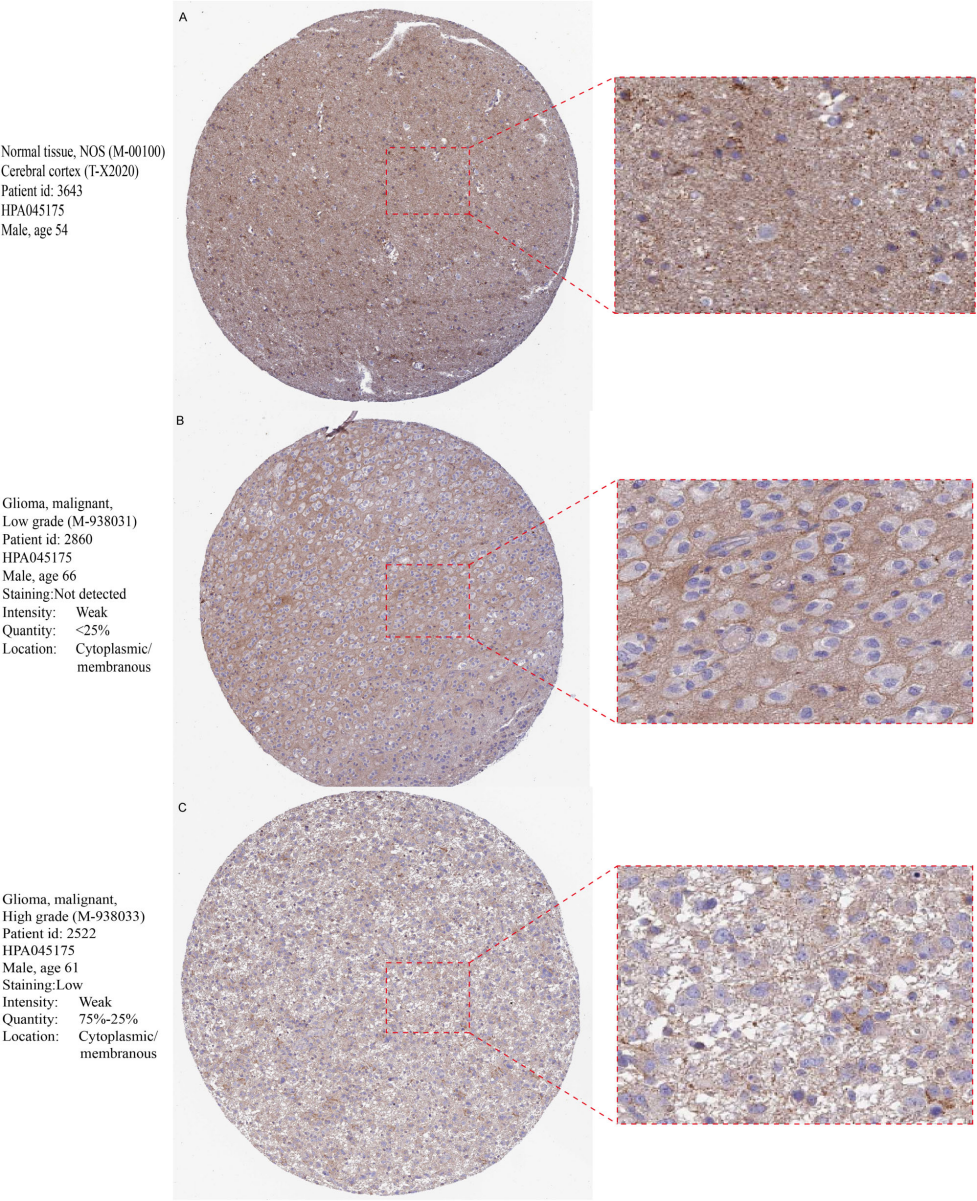
Comparison of immunohistochemical images of ASPHD1 between glioma and normal tissues. The staining intensity of ASPHD1 in normal tissue **(A)** is strong, in low-grade glioma **(B)** it is moderately weak, and in high-grade glioma **(C)** it is very weak.

Additionally, immunohistochemistry indicated that staining for the ASPHD1 antibody was lighter in gliomas, suggesting low expression of this gene in glioma tissues. Furthermore, higher-grade gliomas exhibited lower protein expression than lower-grade gliomas (**Figure 3**).

### Reduced ASPHD1 levels forecast unfavorable outcomes for individuals with glioma

The diagnostic value of ASPHD1 in gliomas was first assessed using ROC (Receiver Operating Characteristic) curves. ASPHD1 expression showed modest but significant ability to distinguish glioma from normal brain tissue, with areas under the curve (AUCs) of 0.646 in the TCGA cohort, 0.710 in the CGGA mRNAseq_325 cohort, and 0.641 in the CGGA mRNAseq_693 cohort (**Figure 2B**). Consistently, time-dependent ROC analysis demonstrated that ASPHD1 alone provided modest discrimination for 3- and 5-year overall survival (OS), with AUCs of 0.683 and 0.753 in the CGGA mRNAseq_325 training cohort and 0.647 and 0.666 in the CGGA mRNAseq_693 validation cohort (**Supplementary Figures S2A, F**). Importantly, when ASPHD1 was incorporated into a multivariable model together with IDH mutation status, the prognostic performance improved, increasing the 3- and 5-year AUCs to 0.774 and 0.832 in CGGA mRNAseq_325 and to 0.755 and 0.748 in CGGA mRNAseq_693, respectively (**Supplementary Figures S2B, G**). In the TCGA dataset, univariate regression analysis revealed that expression level of ASPHD1, whether radiation therapy was performed, age, tumor dimension, tumor location, tumor grade, and tumor pathological type were prognostic indicators in gliomas (**Table 2**). Multivariate regression analysis indicated that low ASPHD1 expression (hazard ratio, 0.999; 95% confidence interval, 0.997–1.000, *P* < 0.05), older age (hazard ratio, 1.060; 95% confidence interval, 1.027–1.093, *P* < 0.001) and higher grade (*P* < 0.001) were independent risk factors of gliomas (**Table 3**). Glioma specimens from TCGA were divided into high- and low-ASPHD1 groups according to the median expression level (H-ASPHD1, n = 335; L-ASPHD1, n = 335). Kaplan–Meier analysis showed that patients with high ASPHD1 expression had significantly longer overall survival (OS) than those with low expression (P < 0.001). Consistent results were obtained in the CGGA mRNAseq_325 and CGGA mRNAseq_693 cohorts, in which high ASPHD1 expression was also associated with a favorable outcome (all P < 0.001; **Figure 2C**). To further evaluate its prognostic value, risk scores derived from the multivariable model incorporating ASPHD1 and conventional covariates were used to stratify patients into four risk groups, yielding clearly separated survival curves in both cohorts (log-rank P < 0.0001; **Supplementary Figures S2D, I**). Decision-curve analysis demonstrated that the model including ASPHD1 together with conventional variables provided the highest net clinical benefit across a wide range of threshold probabilities at 3 years, compared with models based on age and/or IDH alone (**Supplementary Figures S2E, J**). These findings indicate that ASPHD1 contributes independent and incremental prognostic information beyond established glioma markers.

**Table 2:** Results of univariate logistic regression analysis in the glioma cohort in TCGA.

Variables	P-value	HR	95.0% CI	
			Low	High
ASPHD1 mRNA expression (n=674)	<0.001	0.999	0.999	0.999
Age (n=668)	<0.001	1.068	1.057	1.078
Gender (n=674)	0.150	0.828	0.641	1.071
Radiation therapy (n=236)	<0.001	1.896	1.367	2.630
Targeted molecular therapy (n=460)	0.119	1.364	0.923	2.015
Longest dimension (n=225)	0.003	.459	0.272	0.773
Tumor location (n=511)				
Frontal Lobe	0.002			
Parietal Lobe	0.992	0.996	0.476	2.085
Occipital Lobe	0.822	1.176	0.287	4.822
Temporal Lobe	0.001	1.946	1.329	2.849
Posterior Fossa	0.012	4.528	1.398	14.666
WHO grade				
II (n=249)	<0.001			
III (n=265)	<0.001	3.279	2.218	4.848
IV (n=160)	<0.001	18.704	12.558	27.856
Histological type (n=674)				
Astrocytoma	<0.001			
Oligoastrocytoma	0.035	0.604	0.377	0.965
Oligodendroglioma	0.005	0.564	0.378	0.843
Glioblastoma	<0.001	6.575	4.737	9.126

**Table 3:** Multivariate logistic regression analysis of the glioma cohort in TCGA (n=209).

Variables	P-value	HR	95.0% CI	
			Low	High
ASPHD1 mRNA expression	0.026	0.999	0.997	1.000
Age	<0.001	1.060	1.027	1.093
Histological type				
Astrocytoma	0.809			
Oligoastrocytoma	0.669	0.805	0.297	2.178
Oligodendroglioma	0.841	1.101	0.431	2.815
Glioblastoma	0.145	2.414	2.012	2.956
Radiation therapy	0.849	0.903	0.316	2.584
longest dimension	0.017	0.226	0.067	0.766
Tumor location				
Frontal Lobe	0.396			
Parietal Lobe	0.560	1.622	0.319	8.257
Occipital Lobe	0.377	0.344	0.032	3.668
Temporal Lobe	0.127	1.970	0.824	4.708
Posterior Fossa	0.774	1.398	0.142	13.783
WHO grade				
II	<0.001			
III	<0.001	3.017	2.214	3.586
IV	<0.001	10.816	7.882	12.651

In CCGA325, univariate regression analysis showed that age, expression level of ASPHD1, whether radiation therapy was performed, presence or absence of the 1p19q co-deletion, IDH mutation, tumor grade, and whether the tumor was recurrent were prognostic indicators in gliomas (**Table 4**). Multivariate regression analysis indicated that low expression of ASPHD1 (hazard ratio, 0.989; 95% confidence interval, 0.978–0.999, *P* < 0.05), older age (hazard ratio, 1.016; 95% confidence interval, 1.003–1.030, *P* < 0.05), higher grade of glioma, absence of the 1p19q co-deletion (hazard ratio, 0.279; 95% confidence interval, 0.163–0.477, *P* < 0.001), whether recurrence(hazard ratio, 2.593; 95% confidence interval, 1.903–3.533, *P* < 0.001) are independent risk factors (**Table 5**).

**Table 4:** Univariate logistic regression analysis of the glioma cohort in CCGA325.

Variables	P-value	HR	95.0% CI	
			Low	High
ASPHD1 mRNA expression (n=325)	<0.001	0.967	0.956	0.978
Gender (n=325)	0.660	1.063	0.809	1.397
Radiation therapy (n=310)	0.005	0.632	0.457	0.872
Chemotherapy (n=303)	0.014	1.445	1.078	1.937
IDH mutation (n=324)	<0.001	0.355	0.269	0.468
1p/19q codeletion (n=317)	<0.001	0.170	0.104	0.277
Whether MGMT (n=306)	0.178	0.830	0.632	1.089
Age (n=325)	0.000	1.033	1.020	1.046
Whether is recurrent (n=321)	<0.001	2.874	2.160	3.824
WHO grade				
II (n=103)	<0.001			
III (n=79)	<0.001	3.497	2.287	5.348
IV (n=143)	<0.001	8.896	5.992	13.206

**Table 5:** Multivariate logistic regression analysis of the glioma cohort in CCGA325 (n=294).

Variables	P value	HR	95% CI	
			Low	High
ASPHD1 mRNA expression	0.038	0.989	0.978	0.999
WHO grade				
II	<0.001			
III	<0.001	2.646	1.702	4.114
IV	<0.001	4.510	2.905	7.000
IDH mutation	0.310	0.839	0.597	1.178
1p/19q codeletion	0.000	0.279	0.163	0.477
Age	0.013	1.016	1.003	1.030
Whether is recurrent	<0.001	2.593	1.903	3.533

To further quantify the incremental prognostic value of ASPHD1 beyond the conventional age + IDH model, we calculated the category-based net reclassification improvement (NRI) for 3-year OS (**Table 6**). Adding ASPHD1 expression yielded a positive overall NRI in both CGGA cohorts (CGGA mRNAseq_693: NRI = 0.111, 95% CI 0.011–0.191; CGGA mRNAseq_325: NRI = 0.211, 95% CI 0.026–0.355). In both datasets, the improvement was mainly driven by better reclassification of survivors (non-events), with NRI− of 0.137 (95% CI 0.009–0.216) and 0.254 (95% CI 0.036–0.418), reflecting a higher probability that patients who remained alive at 3 years were moved to lower-risk categories (Pr[Down|Ctrl] = 0.178 and 0.254, respectively). By contrast, the NRI+ components for events were close to zero or slightly negative (−0.026 and −0.042), indicating that ASPHD1 had little impact on upward reclassification of deaths. Overall, these results support that incorporating ASPHD1 into an age- and IDH-based model modestly but consistently improves risk discrimination, mainly by reducing overestimation of risk among long-term survivors. The reclassification scatter plots in **Supplementary Figures S2C, H** compare predicted 3-year OS risk from the age + IDH model with that from the age + IDH + ASPHD1 model, showing how inclusion of ASPHD1 shifts individual patients across risk categories in a manner consistent with the positive NRI values.

**Table 6:** Category-based NRI for adding ASPHD1 to age + IDH model at 3-year OS.

Cohort	NRI (95% CI)	NRI+ (events) (95% CI)	NRI− (non-events) (95% CI)	Pr(Up|Case) (95% CI)	Pr(Down|Case) (95% CI)	Pr(Up|Ctrl) (95% CI)	Pr(Down|Ctrl) (95% CI)
CGGA. mRNAseq_693	0.111 (0.011 – 0.191)	-0.026 (−0.057 – 0.046)	0.137 (0.009 – 0.216)	0.019 (0.000 – 0.084)	0.046 (0.013 – 0.072)	0.041 (0.000 – 0.156)	0.178 (0.080 – 0.252)
CGGA. mRNAseq_325	0.211 (0.026 – 0.355)	-0.042 (−0.086 – 0.045)	0.254 (0.036 – 0.418)	0.006 (0.000 – 0.100)	0.048 (0.012 – 0.096)	0.000 (0.000 – 0.131)	0.254 (0.060 – 0.423)

### Analysis of methylation, gene co-expression, and pathway enrichment

**Figure 4:**
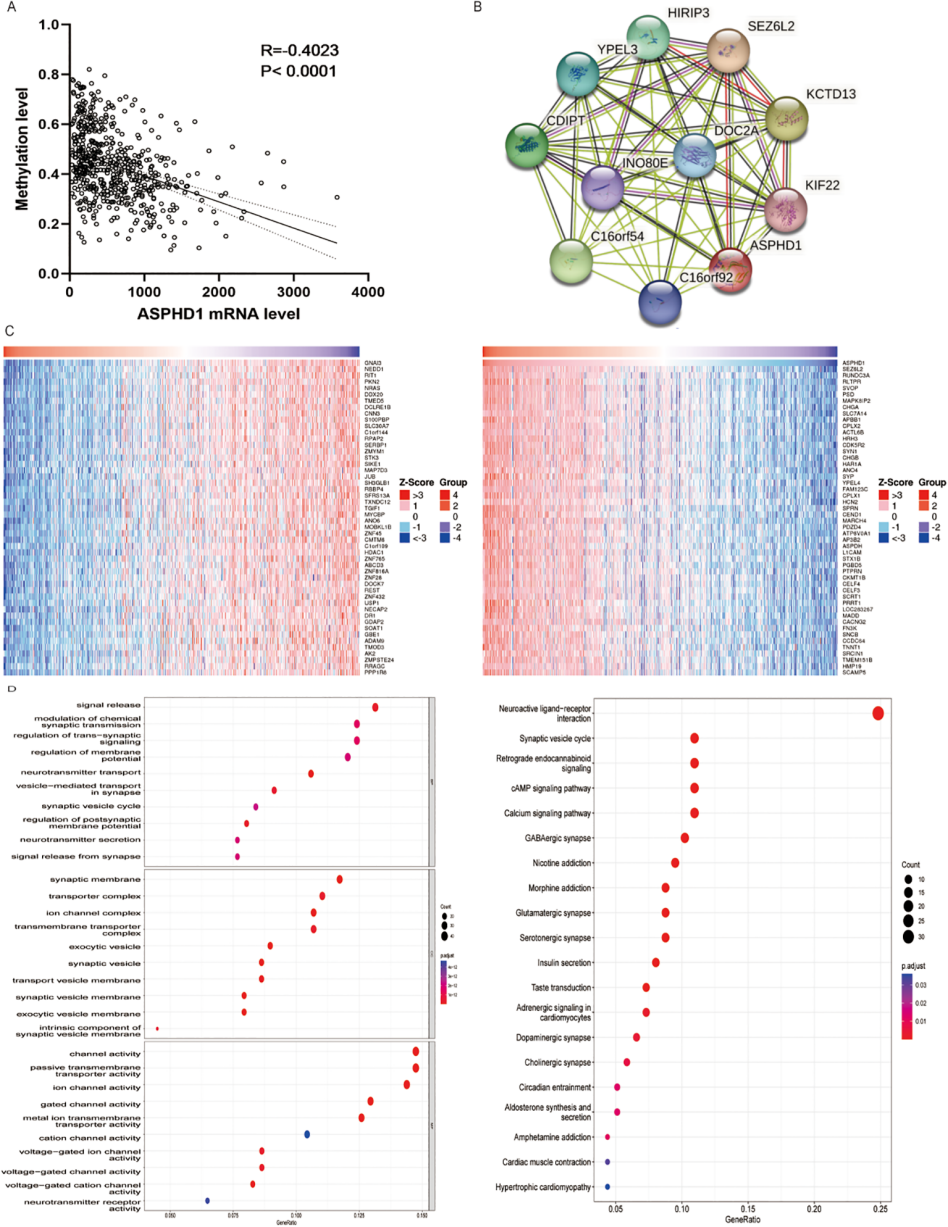
Potential mechanisms of ASPHD1 in glioma. **(A)** Correlation analysis between ASPHD1 methylation and ASPHD1 expression in glioma. **(B)** PPIs of hub genes. **(C)** Heatmap of 50 positively correlating (left) and negatively correlating (right) genes by TISIDB. **(D)** GO analysis (left) and KEGG analysis (right) of ASPHD1 in glioma. Enrichment P values were adjusted for multiple testing using the Benjamini–Hochberg false discovery rate (FDR) method, and only terms with FDR-adjusted P < 0.05 are shown.

A correlation study was conducted between the methylation statuses of the *ASPHD1* gene and its expression levels. As anticipated, an inverse relationship was detected between DNA methylation and *ASPHD1* transcription levels (R = -0.4023, P<0.001) (**Figure 4A**).

The statistically significant differentially expressed genes between glioma and normal tissues are presented as a volcano plot (**Figure 2D**). A total of 3,413 genes were negatively correlated with ASPHD1, as indicated by the red points in the figure, while 3,352 genes were positively correlated with ASPHD1, represented by the green points in the diagram. Heatmaps display the top 50 genes that are either positively or negatively associated with ASPHD1 (**Figure 4C**). Furthermore, two central genes (SEZ6L2 and INO80E) were identified as hub genes pinpointed within the PPI network (**Figure 4B**).

GSEA was employed to perform GO and KEGG pathway enrichment assessments on the entirety of the gene sets that showed either positive or negative co-expression with ASPHD1. The GO enrichment examination revealed that genes concurrently expressed alongside ASPHD1 were primarily concentrated within terms related to biological functions terms such as “presynaptic process”, “modulation of chemical synaptic transmission”, “regulation of trans-synaptic signaling” and “ion channel activity”. Additionally, KEGG enrichment analysis revealed that the genes were primarily linked to pathways concerning “neuroactive substance-receptor engagement”, “cAMP signaling pathway” and “calcium signaling pathway” (**Figure 4D**).

### Pan-cancer analysis shows low expression of ASPHD1 is associated with unfavorable outcomes in various types of tumors

**Figure 5:**
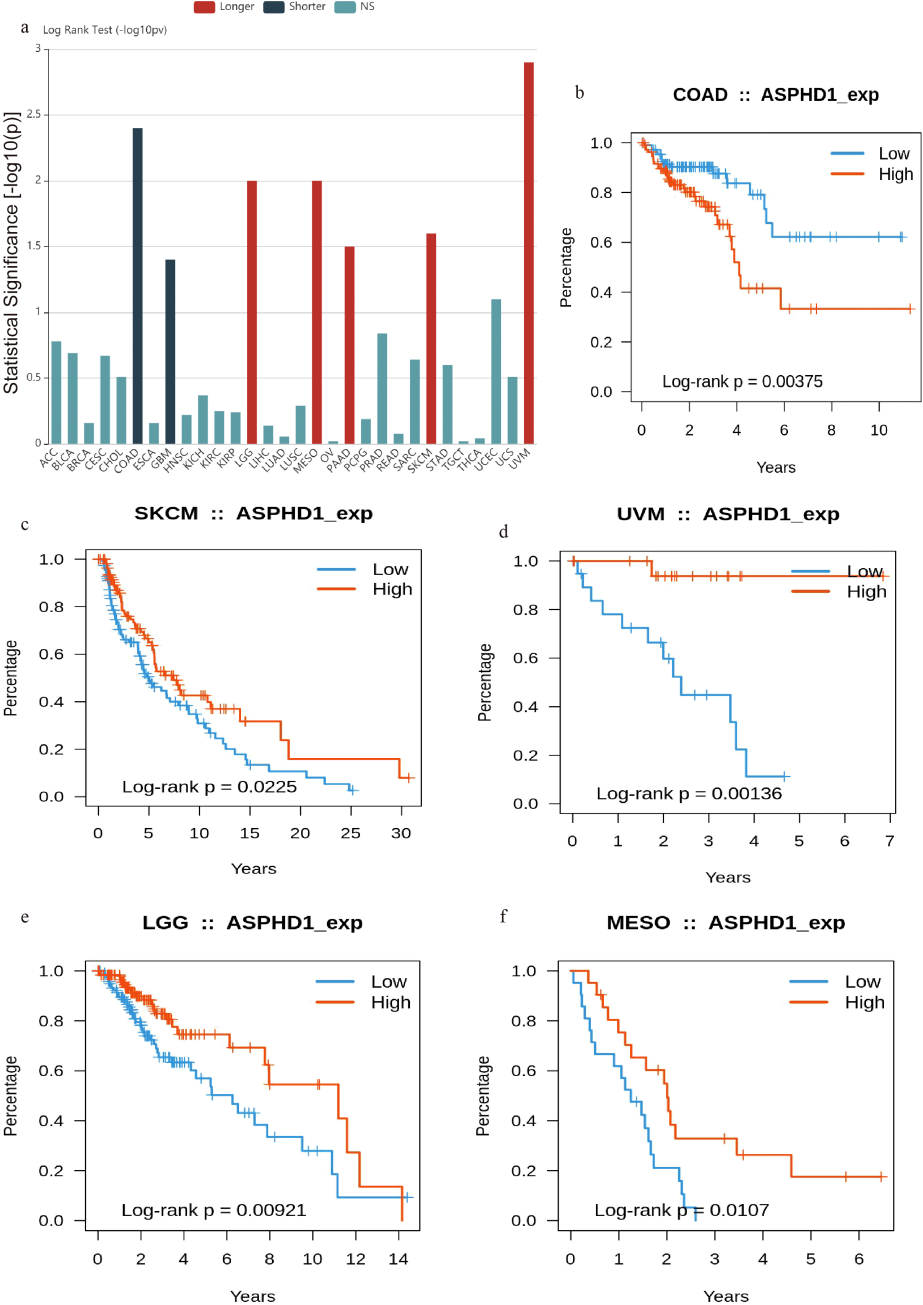
Generalized values of ASPHD1 in pan-cancer. **(a)** Associations between ASPHD1 expression and overall survival across human cancers based on TISIDB. K-M survival analysis of **(b)** COAD, **(c)** SKCM, **(d)** UVM, **(e)** LGG, and **(f)** MESO in the low ASPHD1 and high ASPHD1 groups based on TISIDB and GEPIA. NS: no significance.

To evaluate the potential widespread relevance of ASPHD1, we conducted a range of investigations on its expression across various cancers. Using the GEPIA platform, we assessed the relationship between ASPHD1 levels and the prognosis of various tumors (**Figure 5a**). Among the 30 types of cancer examined, the prognosis of seven cancers was related to ASPHD1 levels. The findings demonstrated that elevated levels of ASPHD1 expression was associated with extended OS in SKCM (**Figure 5c**), UVM (**Figure 5d**), MESO (**Figure 5f**), and low-grade glioma (LGG) (**Figure 5e**). Conversely, higher ASPHD1 expression levels were linked to reduced OS in COAD (**Figure 5b**).

### ASPHD1 level in tumors is related to immunity

**Figure 6:**
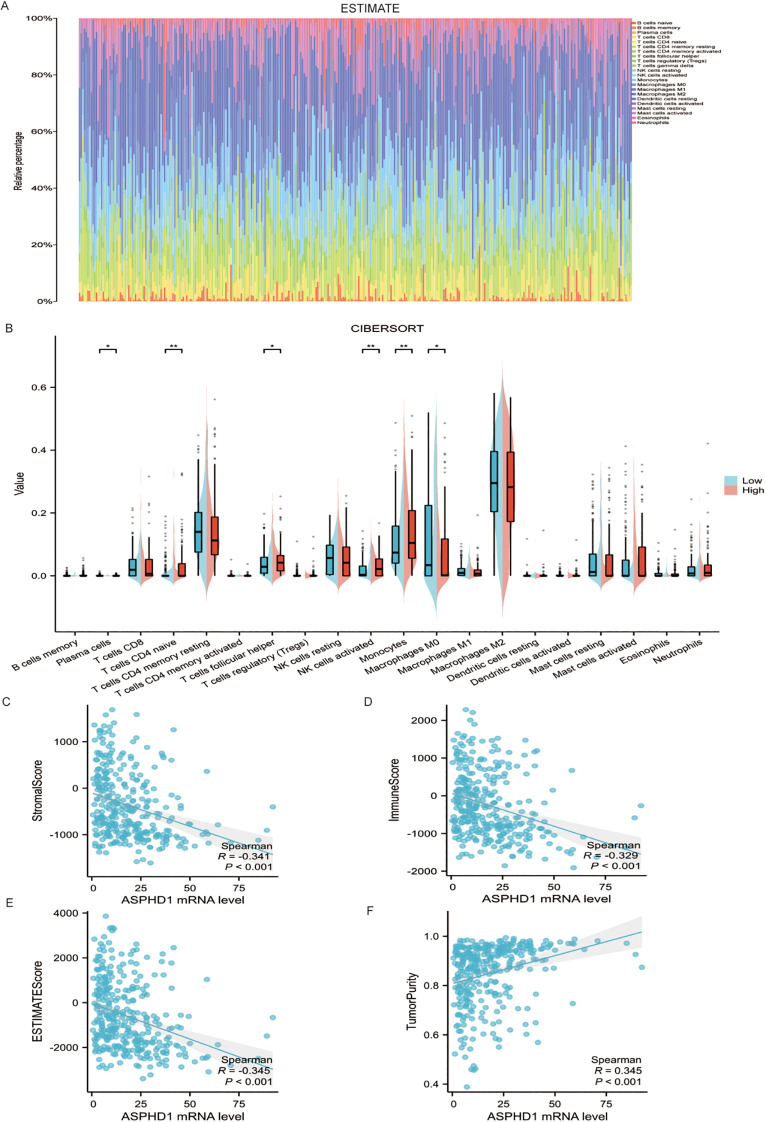
Immune analyses of ASPHD1 in glioma **(A)** ESTIMATE of the proportions of 22 types of immune cells in glioma. **(B)** The distribution differences of 22 types of immune cells in the low and high ASPHD1 expression groups by CIBERSORT. P values for the comparisons of immune cell fractions between ASPHD1-low and ASPHD1-high groups were corrected for multiple testing using the Benjamini–Hochberg FDR procedure, and immune cell types with FDR-adjusted P < 0.05 were considered statistically significant. Relation between ASPHD1 mRNA levels and **(C)** stromal scores, **(D)** immune scores, **(E)** ESTIMATE scores, and **(F)** tumor purity. Correlation P values were also adjusted for multiple testing using the Benjamini–Hochberg FDR method.

ESTIMATE and CIBERSORT analysis demonstrated that gliomas have a higher proportion of macrophage cells and CD4 cells. CIBERSORT showed four immune cell subtypes (CD4+ naïve T cells, monocytes, macrophages M0 and activated NK cells) to be expressed differently between the two groups (**Figures 6A, B**). High-ASPHD1 tumors contained a lower fraction of M0 macrophages than low-ASPHD1 tumors (median 0.12 [IQR 0.06–0.20] vs. 0.23 [IQR 0.14–0.34], P <0.05). By contrast, the proportion of CD4^+^ naïve T cells was higher in the high-ASPHD1 group (median 0.08 [IQR 0.03–0.14] vs. 0.04 [IQR 0.01–0.09], P <0.01). Similar patterns were observed for monocytes (high vs. low: median 0.31 [IQR 0.17–0.39] vs. 0.25 [IQR 0.13–0.31], P <0.01) and activated NK cells (high vs. low: median 0.03 [IQR 0.01–0.06] vs. 0.01 [IQR 0.00–0.03], P <0.01), which were also more abundant in the high-ASPHD1 group. (**Figure 6B**). The association between ASPHD1 expression and immune cell presence was analyzed. There is no significant correlation between ASPHD1 mRNA levels and the stromal score (R=-0.341, P<0.001), immune score (R=-0.329, P<0.001), ESTIMATE score (R=-0.345, P<0.001), and tumor purity (R = 0.345, P<0.001) (**Figures 6C-F**).

### ASPHD1 suppresses the proliferation, migration and invasiveness of glioma cellsASPHD1

**Figure 7:**
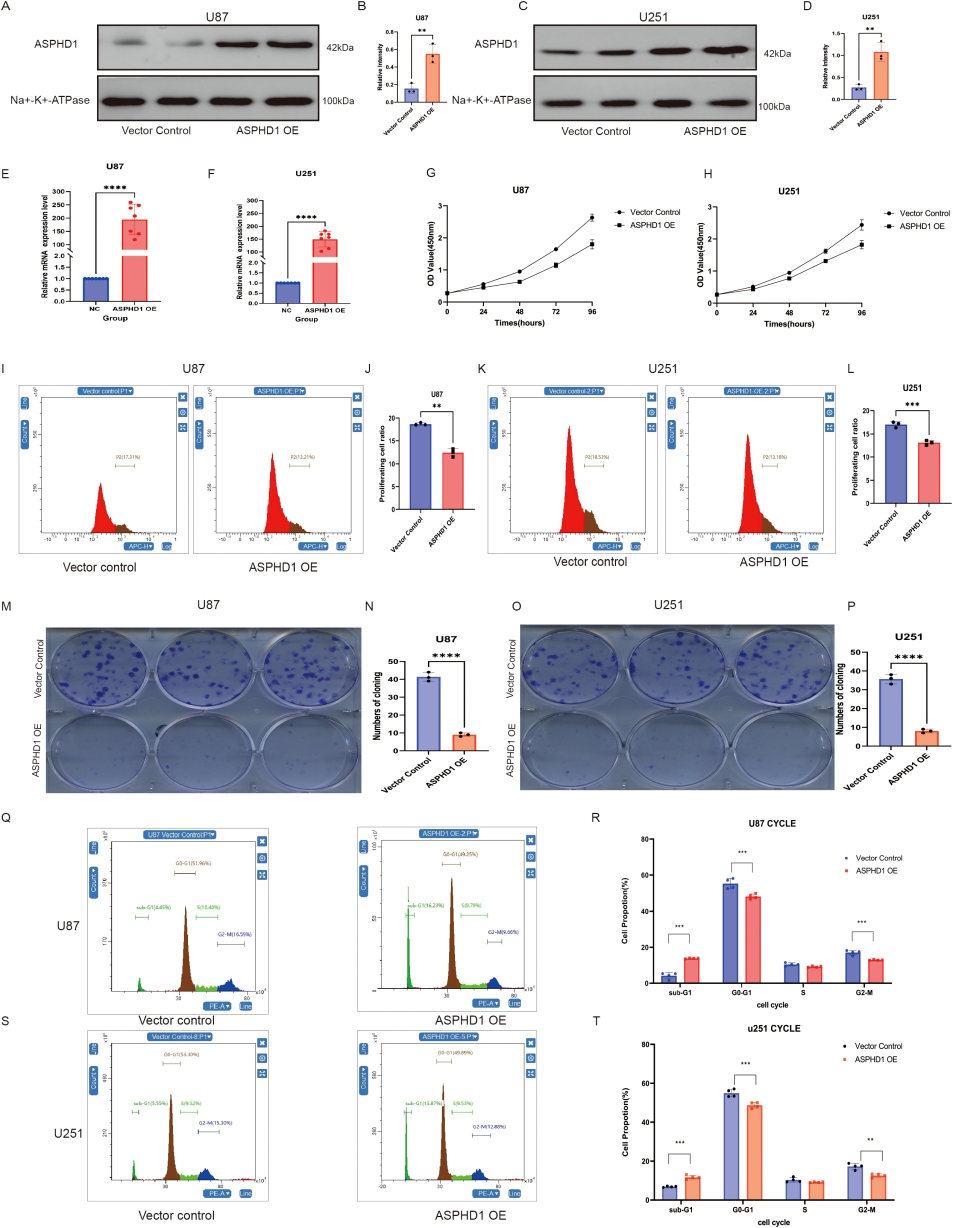
ASPHD1 inhibits the proliferation of glioma cells. **(A, C)** Representative Western blot images showing ASPHD1 protein expression levels in U87 **(A)** and U251 **(C)** cells transfected with empty vector or ASPHD1 overexpression plasmid (ASPHD1 OE). Na+-K+-ATPase was used as a loading control. **(B, D)** Quantification of ASPHD1 protein expression relative to loading control in U87 **(B)** and U251 **(D)** cells (Three independent biological experiments (n = 3), each performed with duplicate wells per condition. **P < 0.01, Vector vs ASPHD1 OE (unpaired two-tailed Student’s t-test).). **(E, F)** qRT-PCR analysis of ASPHD1 mRNA expression levels in U87 **(E)** and U251 **(F)** cells upon ASPHD1 overexpression compared with control (P < 0.0001, Student’s t-test). **(G, H)** CCK-8 assay results showing cell proliferation at indicated time points in U87 **(G)** and U251 **(H)** cells transfected with empty vector or ASPHD1 OE plasmid (P < 0.01, P < 0.001, Student’s t-test). **(I, K)** Representative flow cytometry histograms of EdU incorporation in U87 **(I)** and U251 **(K)** cells transfected with empty vector or ASPHD1 overexpression plasmid (ASPHD1 OE).**(J, L)** Quantification of proliferating cell ratios in U87 **(J)** and U251 **(L)** cells (P < 0.01, P < 0.001, Student’s t-test). **(M, O)** Representative images of colony formation assays in U87 **(M)** and U251 **(O)** cells transfected with empty vector or ASPHD1 overexpression plasmid (ASPHD1 OE).**(N, P)** Quantitative analysis of colony numbers in U87 **(N)** and U251 **(P)** cells (P < 0.0001, Student’s t-test). **(Q, S)** Representative flow cytometry histograms showing the cell cycle distribution (sub-G1, G0/G1, S, and G2/M phases) of U87 **(Q)** and U251 **(S)** cells transfected with empty vector or ASPHD1 overexpression plasmid (ASPHD1 OE). **(R, T)** Quantitative analyses of the cell proportions in each phase of the cell cycle in U87 **(R)** and U251 **(T)** cells. (P < 0.01, P < 0.001, Student’s t-test). Data represent mean ± SD of three independent experiments. **P < 0.01; ***P < 0.001; ****P < 0.0001.

Finally, we explored the functional roles of ASPHD1 in glioma, using the U251 and U87 cell models. These models were genetically modified to overexpress ASPHD1. Western blot analysis confirmed the successful overexpression of ASPHD1 protein in both U87 and U251 glioma cells. As shown in **Figures 7A, C**, compared with the vector control group, the protein expression levels of ASPHD1 were significantly increased in U87 (P < 0.01, **Figure 7B**) and U251 (P < 0.01, **Figure 7D**) cells transfected with the ASPHD1 overexpression plasmid (ASPHD1 OE). Consistently, comparison of parental and vector-transduced cells showed no obvious differences in ASPHD1 protein levels or proliferation in either U87 or U251 lines (**Supplementary Figures S1B, C**); therefore, vector cells were used as the main control group in subsequent overexpression experiments. Additionally, qRT-PCR analysis revealed a robust increase in ASPHD1 mRNA expression in ASPHD1-overexpressing cells compared to vector control in both U87 (P < 0.0001, **Figure 7E**) and U251 cells (P < 0.0001, **Figure 7F**). The effect of ASPHD1 overexpression on cell proliferation was further confirmed using the CCK-8 assay. As shown in **Figures 7G, H**, the proliferation rate of both U87 and U251 cells significantly decreased upon ASPHD1 overexpression compared with vector control cells at different time points. The EdU proliferation assay revealed that overexpression of ASPHD1 significantly reduced the proliferation of both U87 and U251 glioma cells compared to the vector control groups. Specifically, the proliferating cell ratio significantly decreased from approximately 17.31% (Vector control) to 13.21% (ASPHD1 OE) in U87 cells (P < 0.01) (**Figures 7I, J**). Similarly, in U251 cells, the proliferating cell ratio was significantly reduced from 18.39% (Vector control) to 13.18% (ASPHD1 OE) (P < 0.001) (**Figures 7K, L**). Colony formation assays were conducted to evaluate the long-term effects of ASPHD1 overexpression on glioma cell growth. As shown in **Figures 7M, O**, ASPHD1 overexpression significantly reduced colony-forming capacity compared with vector control groups in both U87 and U251 cell lines. Quantitative analysis revealed that ASPHD1 overexpression markedly decreased the colony numbers from approximately 40 colonies to fewer than 10 colonies in U87 cells (P < 0.0001, **Figure 7N**), and similarly from approximately 35 colonies to fewer than 10 colonies in U251 cells (P < 0.0001, **Figure 7P**). These findings suggest that ASPHD1 significantly inhibits glioma cell clonogenic survival. These results suggest a robust inhibitory effect of ASPHD1 on glioma cell proliferation.

**Figure 8:**
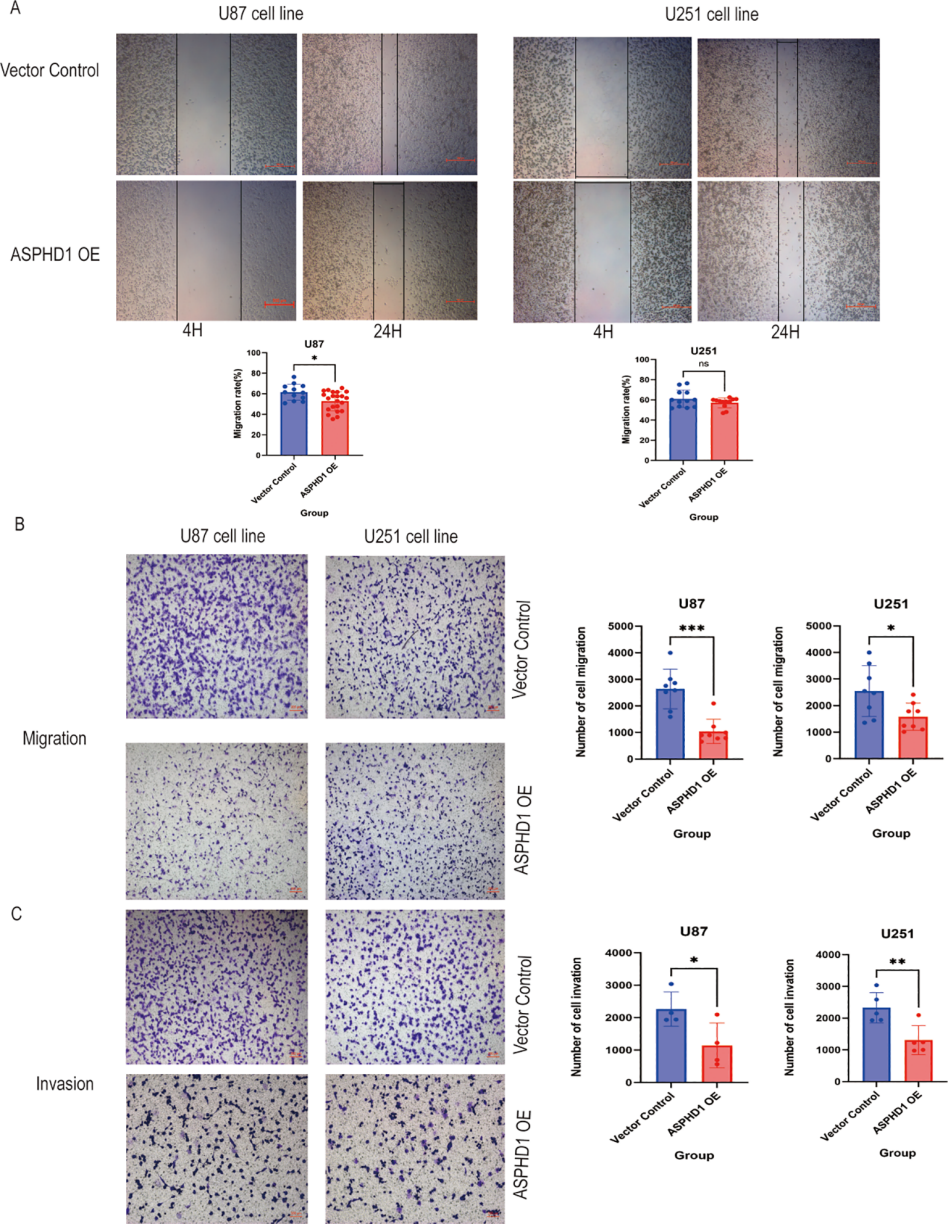
ASPHD1 inhibits the invasiveness of glioma cells. **(A)** Illustrations of cell scratch wounds experiment. After 4 hours of cell adhesion, create a scratch using a pipette tip. After 24 hours, the scratch wounds were partly covered by U87 and U251 glioma cells. The bar chart below summarizes the difference in migration abilities between the overexpression group and the control group. Cell migration ability is represented by the migration rate, which is calculated as (migrated distance - initial distance) / initial distance. **(B)** Transwell migration assay showing that the ability of U87 and U251 glioma cells to migrate across a membrane. The bar chart on the right shows the difference in migration ability between the overexpression group and the control group. **(C)** Transwell invation assay showing that the ability of U87 and U251 glioma cells to invade across a Matrigel-coated membrane. The bar chart on the right shows the difference in invasive ability between the overexpression group and the control group. All of the experiments were performed at least three times. Data presented are the mean ± SD; ns. p > 0.05, *p < 0.05, **p < 0.01, ***p < 0.001. compared with control using Student t-test.

Flow cytometric analysis revealed that ASPHD1 overexpression significantly altered the cell cycle distribution of both U87 and U251 glioma cells (**Figures 7Q-T**). Specifically, ASPHD1 overexpression significantly increased the proportion of cells in the sub-G1 phase (P < 0.001), which is consistent with an increase in cell death. Additionally, ASPHD1 overexpression resulted in an increased proportion of cells in the G0/G1 phase, accompanied by a significant decrease in cells in the G2/M phase in both U87 (P < 0.001) and U251 cells (P < 0.01),whereas no significant differences were observed in the S phase. These findings indicate that ASPHD1 overexpression causes G0/G1 cell cycle arrest and reduces the proliferative fraction of glioma cells, in line with the reduced proliferation observed in the CCK-8, colony formation, and EdU assays. To obtain complementary loss-of-function evidence, we generated U251 cells with stable shRNA-mediated knockdown of ASPHD1. Western blotting confirmed that ASPHD1 protein levels were markedly reduced in shASPHD1 cells compared with parental and shNC controls (**Supplementary Figure S3A**). EdU staining showed that ASPHD1 knockdown significantly increased the proportion of proliferating cells relative to both parental and shNC groups (**Supplementary Figures S3B, D**). Wound-healing assays further demonstrated that shASPHD1 cells closed the scratch more rapidly than control cells, and the quantified migration rate was significantly higher in the shASPHD1 group (**Supplementary Figures S3C, E**). Because parental and shNC cells displayed comparable ASPHD1 expression and proliferation, shNC cells were used as the main control group in subsequent knockdown experiments. These reciprocal findings from gain- and loss-of-function models support a specific role for endogenous ASPHD1 in restraining glioma cell proliferation and migration. We investigated the impact of ASPHD1 overexpression on cell migration and invasion capabilities. In scratch assays, we found that ASPHD1 overexpression could inhibit migration in the U87 cell line; however, this effect was not observed in the U251 cell line (**Figure 8A**). We further validated these findings with Transwell migration assays, noting a reduction in migration in both cell lines, although the effect was less pronounced in the U251 line, likely due to differences between the cell lines (**Figure 8B**). Finally, we conducted transwell invasion assays, and the results indicated that ASPHD1 overexpression could inhibit invasion in both U87 and U251 cells (**Figure 8C**).

### ASPHD1 overexpression inhibited tumor growth *in vivo* in a subcutaneous U87 xenograft model

**Figure 9:**
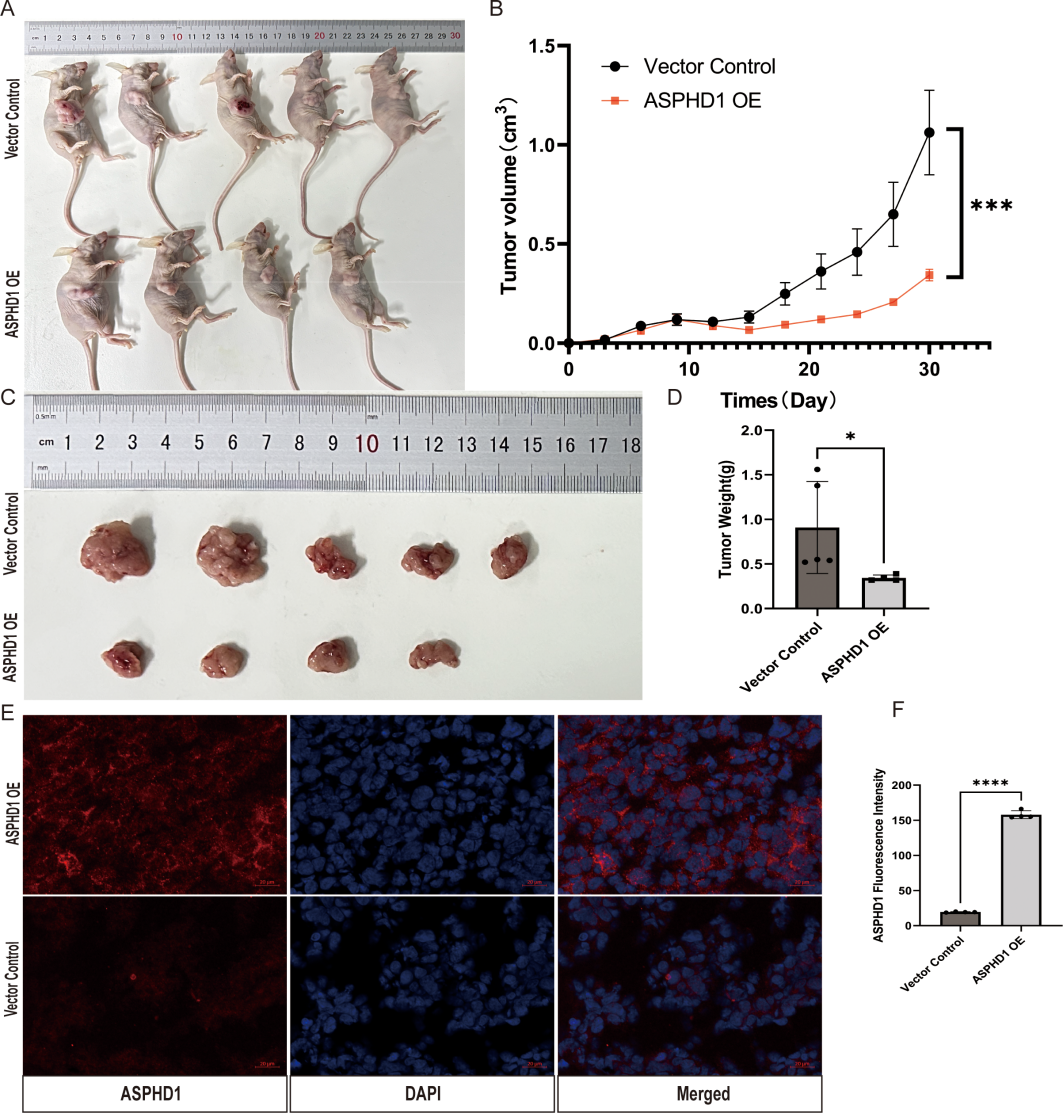
ASPHD1 overexpression suppresses tumor growth and invasiveness in a glioma xenograft model. **(A)** Subcutaneous tumor transplantation in BALB/c-nu femalemice using U87 cells with ASPHD1 OE(N = 4) or an empty vector (n = 5, left). **(B)** Growth curve of the tumors in the ASPHD1 OE and vector groups (right). Tumor volume was calculated using the formula V = ab^2^/2, where a and b are the tumor length and width, respectively. **(C, D)** Comparison of the tumor volume and weight between the ASPHD1 OE and vector groups. **(E)** Representative immunofluorescence images of ASPHD1 (red) and nuclei (DAPI, blue) in xenograft tumors derived from vector control (Vector) and ASPHD1-overexpressing (ASPHD1 OE) U87 cells. All images were acquired under identical exposure and gain settings. Scale bar, 20 μm. **(F)** Quantification of ASPHD1 fluorescence intensity in xenograft tumors. Data presented are the mean ± SD; ns. p > 0.05, *p < 0.05, ***p < 0.001, ****p < 0.0001. compared with control using Student t-test.

To determine whether ASPHD1 OE suppressed glioma cell–derived tumor growth *in vivo*, we established stable U87 cell lines by integrating an ASPHD1 OE vector or the control vector. These modified cell lines were subcutaneously injected into the right flank of BALB/c-nu mice to generate subcutaneous xenograft tumors in separate groups. After 30 days, the volume and weight of the xenograft tumors were significantly lower in the ASPHD1 OE group than those in the control group (**Figures 9A–D**). The immunofluorescence results confirmed that xenografts in the ASPHD1 OE group exhibited elevated ASPHD1 expression levels compared with control tumors (**Figures 9E, F**), consistent with the reduced tumor burden observed in mice bearing ASPHD1-overexpressing xenografts.

### ASHPD1 overexpression promotes neuronal differentiation in glioma cells

**Figure 10:**
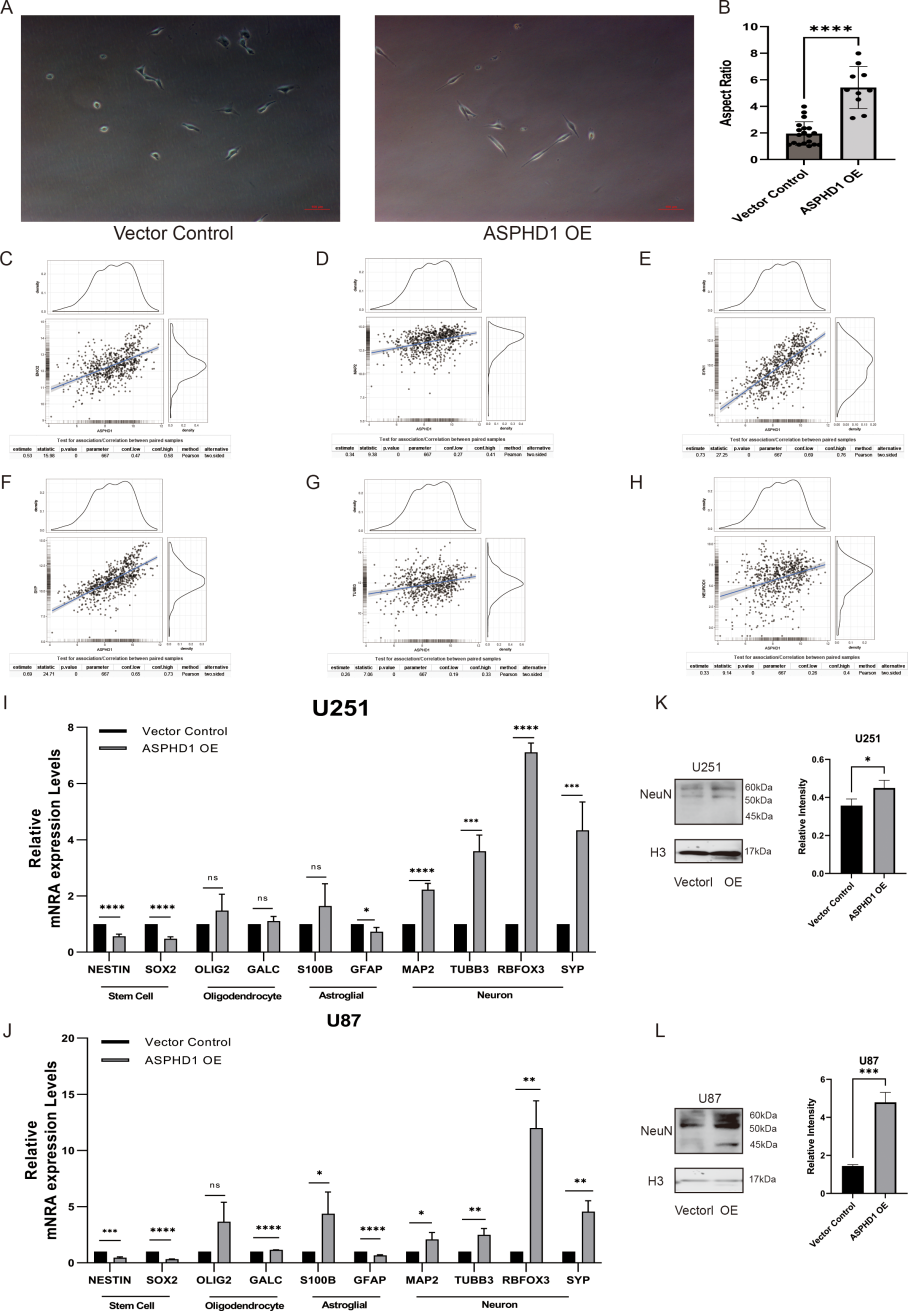
ASPHD1 overexpression induces neuronal differentiation and morphological changes in glioma cells. **(A)** Representative phase-contrast images of U87 cells transduced with vector control or ASPHD1-overexpressing (OE) plasmids. ASPHD1 OE cells exhibit more elongated and polarized morphology. Scale bar, 50 μm.**(B)** Quantification of cell aspect ratio in U87 cells (n=10 fields per group). Data are presented as mean ± SD. ****p < 0.0001 by unpaired two-tailed Student's t-test.**(C–H)** Correlation analyses between ASPHD1 and neuronal differentiation markers (ENO2, MAP2, TUBB3, RBFOX3, SYP and NeuroD1) in TCGA glioma cohorts. Scatter plots depict Pearson correlation coefficients and significance levels. **(I, J)** qPCR analysis of relative mRNA expression levels of stem cell (NESTIN, SOX2), oligodendrocyte (OLIG2, GALC), astroglial (S100B, GFAP), and neuronal (MAP2, TUBB3, RBFOX3, SYP) markers in U251 **(I)** and U87 **(J)** cells transduced with control or ASPHD1 OE vectors. Western blot analysis of NeuN expression in U251 **(K)** and U87 **(L)** cells following ASPHD1 overexpression (OE) or vector control transfection. Histone H3 (H3) was used as a loading control. Quantification of NeuN band intensity is shown on the right. Data represent mean ± SD (n=3). ns, not significant; *p < 0.05; **p < 0.01; ***p < 0.001; ****p < 0.0001 by unpaired two-tailed Student's t-test.

**Figure 11:**
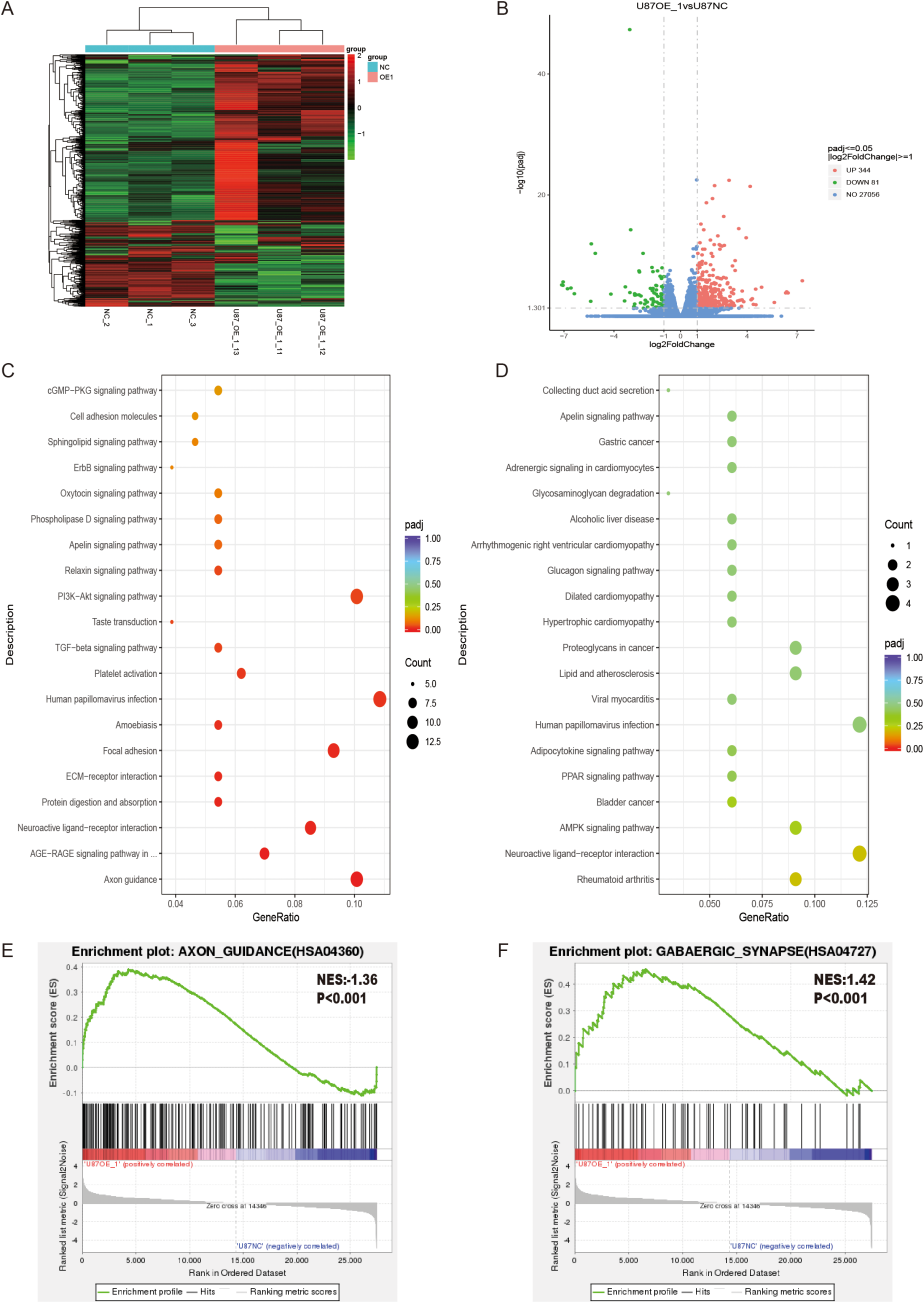
Transcriptomic analysis demonstrates that ASPHD1 overexpression promotes neuronal differentiation pathways in glioma cells. **(A)** Heatmap of hierarchical clustering illustrating differential gene expression between ASPHD1-overexpressing (OE) and negative control (NC) groups. **(B)** Volcano plot representing significantly differentially expressed genes (red dots indicate significantly upregulated genes, green dots indicate significantly downregulated genes, and blue dots represent non-significant genes). **(C, D)** KEGG pathway enrichment bubble plots displaying significantly enriched pathways for upregulated **(C)** and downregulated **(D)** genes, respectively. Color represents adjusted p-values, and bubble size corresponds to gene count. **(E, F)** Gene Set Enrichment Analysis (GSEA) plots depicting enrichment in Axon guidance **(E)** and GABAergic synapse **(F)** pathways.

As mentioned above, GO and KEGG enrichment analyses identified pathways such as electrophysiology and neuronal axon development, suggesting that ASPHD1 may be involved in neuronal differentiation. As shown in the phase-contrast images (**Figure 10A**, **Supplementary Figure S1D**), parental, vector-control, and ASPHD1 OE U87 cells were examined on Day2, day 3 and day 5 after seeding, ASPHD1 OE U87 cells displayed a more elongated, neuron-like morphology than parental and vector-control cells. Phase-contrast microscopy revealed that ASPHD1 OE U87 cells exhibited a more elongated and polarized morphology compared to control cells. Quantitative analysis of cell shape on Day2, day 3 showed that the aspect ratio of U87 cells was significantly increased in the ASPHD1 OE group compared with both parental and vector-control cells (**Figure 10B**, **Supplementary Figure S1E**, P < 0.0001), suggesting enhanced neurite-like outgrowth and cellular elongation. To assess whether these morphological changes were associated with a shift in lineage marker expression, we analyzed stem cell, oligodendrocyte, astroglial, and neuronal lineage markers by qPCR in both U251 and U87 cells. ASPHD1 overexpression significantly decreased the expression of stem cell markers (NESTIN, SOX2), while inducing a robust upregulation of neuronal markers (MAP2, TUBB3, RBFOX3, SYP) in both cell lines (**Figures 10I, J**). Markers for astroglial (GFAP, S100B) and oligodendrocyte (OLIG2, GALC) lineages showed minimal or inconsistent changes. These findings suggest that ASPHD1 OE drives glioma cells towards neuronal differentiation. Furthermore, analysis of TCGA glioma datasets revealed significant positive correlations between ASPHD1 expression and neuronal differentiation markers, including ENO2, MAP2, TUBB3, RBFOX3, SYP and NeuroD1 (**Figures 10C–H**), supporting the clinical relevance of ASPHD1 in promoting neuronal lineage commitment in gliomas. To investigate whether ASPHD1 overexpression promotes neuronal differentiation in glioma cells, we assessed the expression of NeuN, a well-established neuronal marker, in U251 and U87 cells. Western blot analysis revealed a significant upregulation of NeuN protein levels in ASPHD1-overexpressing cells compared to vector controls. In U251 cells, ASPHD1 overexpression moderately increased NeuN expression (P < 0.05) (**Figure 10K**), whereas in U87 cells a more pronounced elevation was observed (P < 0.001) (**Figure 10L**). At the molecular level, GFAP protein expression was reduced in ASPHD1-overexpressing cells and increased upon ASPHD1 knockdown (**Supplementary Figure S3E**). Taken together, these reciprocal changes in NeuN and GFAP expression suggest that ASPHD1 promotes neuronal-like differentiation in glioma cells. To verify whether ASPHD1 overexpression promotes tumor cell differentiation, we performed transcriptome sequencing analysis. Hierarchical clustering heatmap revealed significant differences in gene expression profiles between ASPHD1-overexpressing cells (OE) and control cells (NC) (**Figure 11A**). Differential gene expression analysis identified 344 significantly upregulated genes and 81 significantly downregulated genes (**Figure 11B**). KEGG enrichment analysis indicated that the differentially upregulated genes were significantly enriched in neuronal differentiation-related pathways, including Axon guidance, AGE-RAGE signaling pathway, Neuroactive ligand-receptor interaction, and ECM-receptor interaction (**Figure 11C**). In contrast, downregulated genes were predominantly enriched in pathways related to metabolism and cardiomyopathy, such as Rheumatoid arthritis, AMPK signaling pathway, and Adipocytokine signaling pathway (**Figure 11D**). Gene set enrichment analysis (GSEA) further supported that ASPHD1 overexpression was positively associated with neuronal differentiation pathways, notably the Axon guidance pathway (**Figure 11E**) and the GABAergic synapse pathway (**Figure 11F**).

In U251 glioma cells we next examined whether ASPHD1 affects basic electrophysiological properties and Ca²^+^ signaling (**Supplementary Figure S3**). Whole-cell patch-clamp recordings showed that ASPHD1 overexpression markedly hyperpolarized the resting membrane potential compared with vector control cells: vector-infected cells stabilized around −40 to −50 mV, whereas ASPHD1-overexpressing cells stabilized around −60 to −80 mV (**Supplementary Figures S3A, B**). Step current injections did not elicit overshooting action potentials in either condition, indicating that U251 cells remain non-spiking but that ASPHD1 shifts them towards a more negative resting state.

To obtain an orthogonal functional readout, we performed Fluo-4 AM–based calcium imaging in response to acetylcholine (ACh) stimulation. ASPHD1-overexpressing cells exhibited robust and reproducible ACh-evoked Ca²^+^ transients, with large increases in ΔF/F_0_ in a substantial fraction of cells, whereas vector control cells showed only modest changes in fluorescence (**Supplementary Figures S3C, D**, upper panels). Conversely, shRNA-mediated knockdown of ASPHD1 in U251 cells strongly attenuated ACh-induced Ca²^+^ responses compared with shNC cells, which displayed clear oscillatory Ca²^+^ signals under the same stimulation paradigm (**Supplementary Figures S3C, D**, lower panels). These data support the notion that ASPHD1 promotes a neuron-like, less astrocytic differentiation state in glioma cells and enhances their depolarization-evoked Ca²^+^signaling.

## Discussion

**Figure 12:**
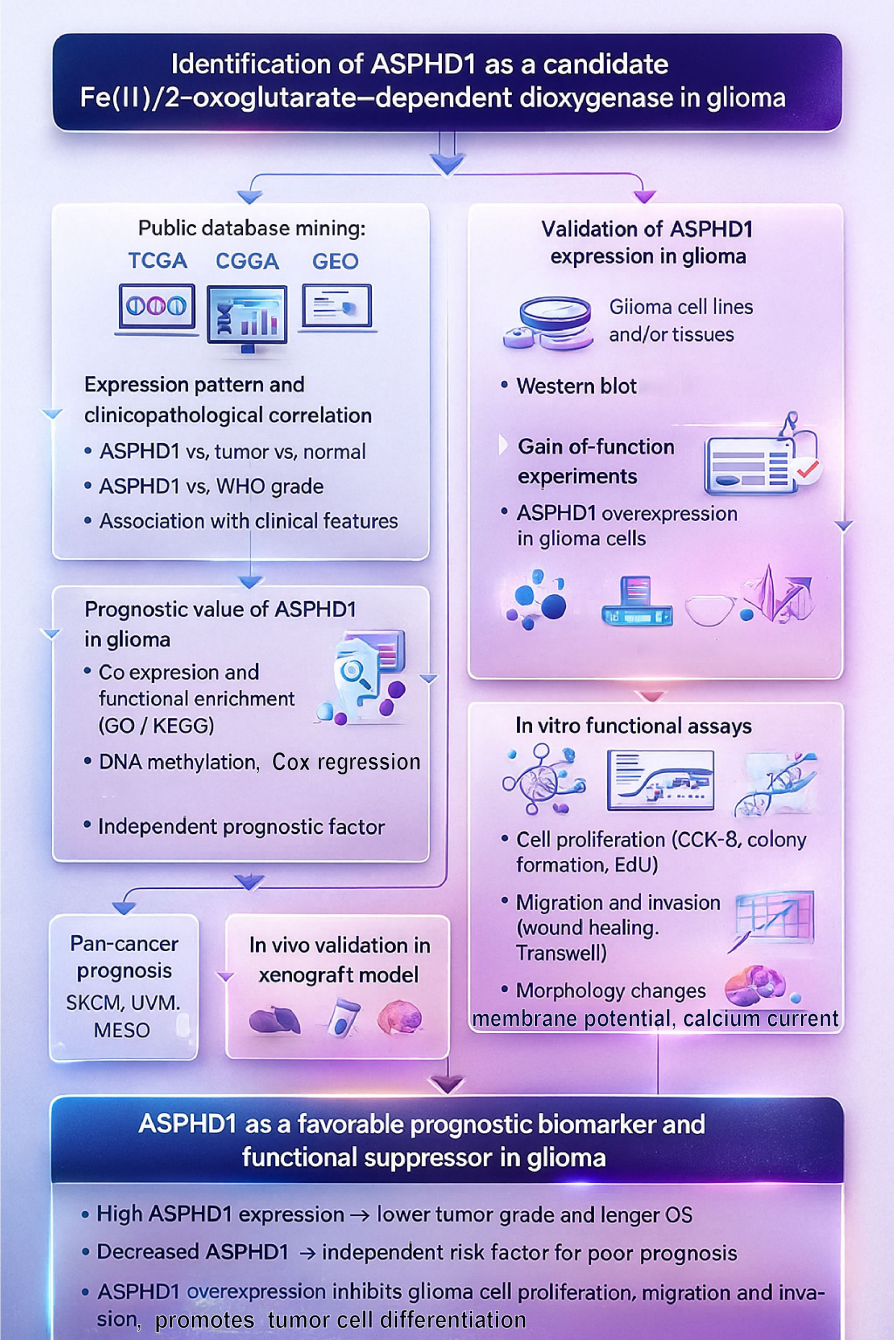
Schematic overview of the study design and main findings. ASPHD1 was first identified as a candidate Fe(II)/2-oxoglutarate–dependent dioxygenase in glioma by mining public databases (TCGA, CGGA, GEO) for expression patterns, clinicopathological correlations, and prognostic value, including co-expression/GO–KEGG enrichment, DNA methylation, and Cox regression analyses. ASPHD1 expression and function were then validated in glioma cell lines and tissues using gain-of-function approaches and *in vitro* assays of proliferation, migration, invasion, morphology, resting membrane potential, and calcium responses, as well as in a subcutaneous xenograft model. Together, these data support ASPHD1 as a favorable prognostic biomarker and functional suppressor in glioma.

Glioma represents a highly common primary aggressive and fatal brain neoplasm. However, the molecular mechanisms are still not well understood. In our study, we initially showed reduced levels of ASPHD1 in glioma tissue compared to normal tissues, followed by evaluating its diagnostic and prognostic value through extensive computational biology analysis. To delve deeper into the possible role of ASPHD1, we pinpointed genes associated with ASPHD1 expression and conducted functional enrichment analyses. KEGG pathway and GO analyses showed an abundance of genes involved in neuronal synaptic signaling, ion-channel activity, and calcium signaling, pointing to a potential link with neuronal and synaptic function. Our research additionally indicates that ASPHD1 expression does not show an association with tumor purity or immune infiltration. Four immune cell subtypes (CD4+ naïve T cells, monocytes, macrophages M0 and activated NK cells) to be expressed differently between the two groups (H-ASPHD1 and L-ASPHD1), so immune infiltration may play a role between ASPHD1 and glioma. Via analysis related to methylation, we established that DNA methylation could potentially regulate the expression of the ASPHD1 gene. Finally, our functional data support a tumor-suppressive role of ASPHD1 and indicate that it promotes a neuron-like differentiated state in glioma cells (**Figure 12**).

The results of this research emphasize the significance of ASPHD1 for clinical indicator use, underscore its capability as a prognostic indicator for glioma patient outcomes. Although the discrimination of ASPHD1 alone is only moderate (AUCs around 0.65–0.75), integrating ASPHD1 expression with canonical clinical markers such as age and IDH mutation consistently improved model performance and net clinical benefit in two independent CGGA cohorts, supporting its role as a complementary prognostic biomarker rather than a stand-alone test. The intrinsic mechanism of ASPHD1 in cancer onset and advancement is complex and not well understood. KEGG pathway mapping indicates the pinpointed genes are primarily engaged in processes related to “neurotransmitter receptor interactions”, “cAMP signaling pathway” and “calcium signaling pathway”. Pal et al. recently demonstrated that a CT-CALCR signaling axis, as a disrupted mechanism of neural signaling communication, constitutes a significant pathway for tumor suppression in glioma, and its malfunctioning is linked to unfavorable outcomes in patients (19). Losada-Pérez et al. discovered that presynaptic and postsynaptic proteins contribute to the advancement and fatality of glioma (20). Chhipa et al. found that AMP kinase enhances glioma bioenergetics and expansion through the AMPK-CREB1 process, involving the regulatory proteins HIF1α and GABPA (21). Activation of the glutamatergic receptors can drive glioma progression by increasing intracellular Ca^2+^ (22). More and more data indicate that Ca^2+^ could play a crucial role in the regulation of tumor development in glioma (23). It has been demonstrated that malignant gliomas secrete glutamate, which glioma cells then utilize to facilitate migration by triggering Ca2+-permeable AMPA receptors (24–26). Therefore, further research may involve investigation of the specific mechanism by which ASPHD1 regulates glioma through these signaling pathways above.

One limitation of the current study is that the fundamental biochemical and cellular functions of ASPHD1 remain incompletely understood, even though our data support its role as a new prognostic indicator for glioma. This gap in knowledge may affect the reliability of using ASPHD1 as a prognostic tool, and our findings should therefore be interpreted with caution until its biological roles are more fully elucidated. The precise molecular mechanisms and signaling pathways downstream of ASPHD1 were not identified in this work. In addition, our bioinformatic analyses were retrospective and based on publicly available cohorts with heterogeneous clinical management, and prospective validation in well-annotated series will be important before clinical implementation. As a potential resolution to these limitations, future studies will focus on identifying ASPHD1-interacting partners and delineating the signaling pathways through which it regulates differentiation and proliferation.

Our *in vivo* findings were obtained in a subcutaneous U87 xenograft model rather than an orthotopic intracranial glioma model. Subcutaneous xenografts do not fully recapitulate the brain microenvironment, including the blood–brain barrier, brain-specific extracellular matrix, and interactions with resident glial and immune cells. Therefore, the *in vivo* effects of ASPHD1 on glioma progression observed here should be interpreted with caution, and future studies incorporating orthotopic intracranial models will be important to validate these findings under more physiologically relevant conditions.

## Conclusions

ASPHD1 appears to be a favorable prognostic biomarker in glioma and may have potential diagnostic value, although its performance as a stand-alone marker is modest and requires further validation. High ASPHD1 expression is associated with less aggressive glioma phenotypes and a more neuron-like, differentiated state, possibly mediated through synaptic signaling, ion-channel, and calcium-related pathways. In addition, pan-cancer analyses suggest that ASPHD1 expression is associated with prognosis in several other tumor types.

## Data Availability

The datasets presented in this study can be found in online repositories. The names of the repository/repositories and accession number(s) can be found in the article/**Supplementary Material**.
