# Supplementary figures and images for: ASPHD1 Is a tumor-suppressive and prognostic marker in glioma
Source: Front Oncol. 2026 Jan 2;15:1694116. doi: 10.3389/fonc.2025.1694116 (PMC12807891; doi:10.3389/fonc.2025.1694116)

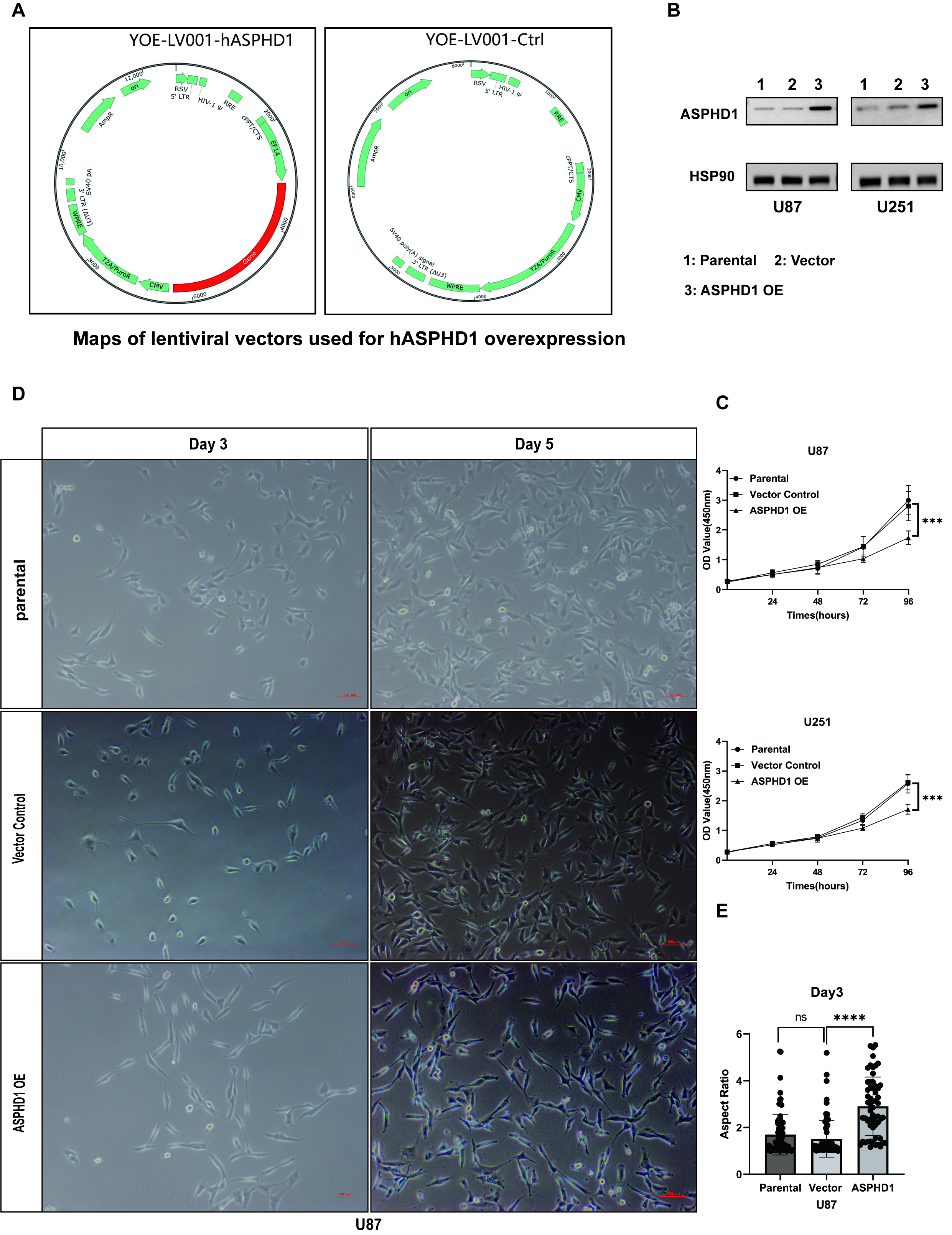

Supplement: Supplementary file 1 [file Image1.tif]

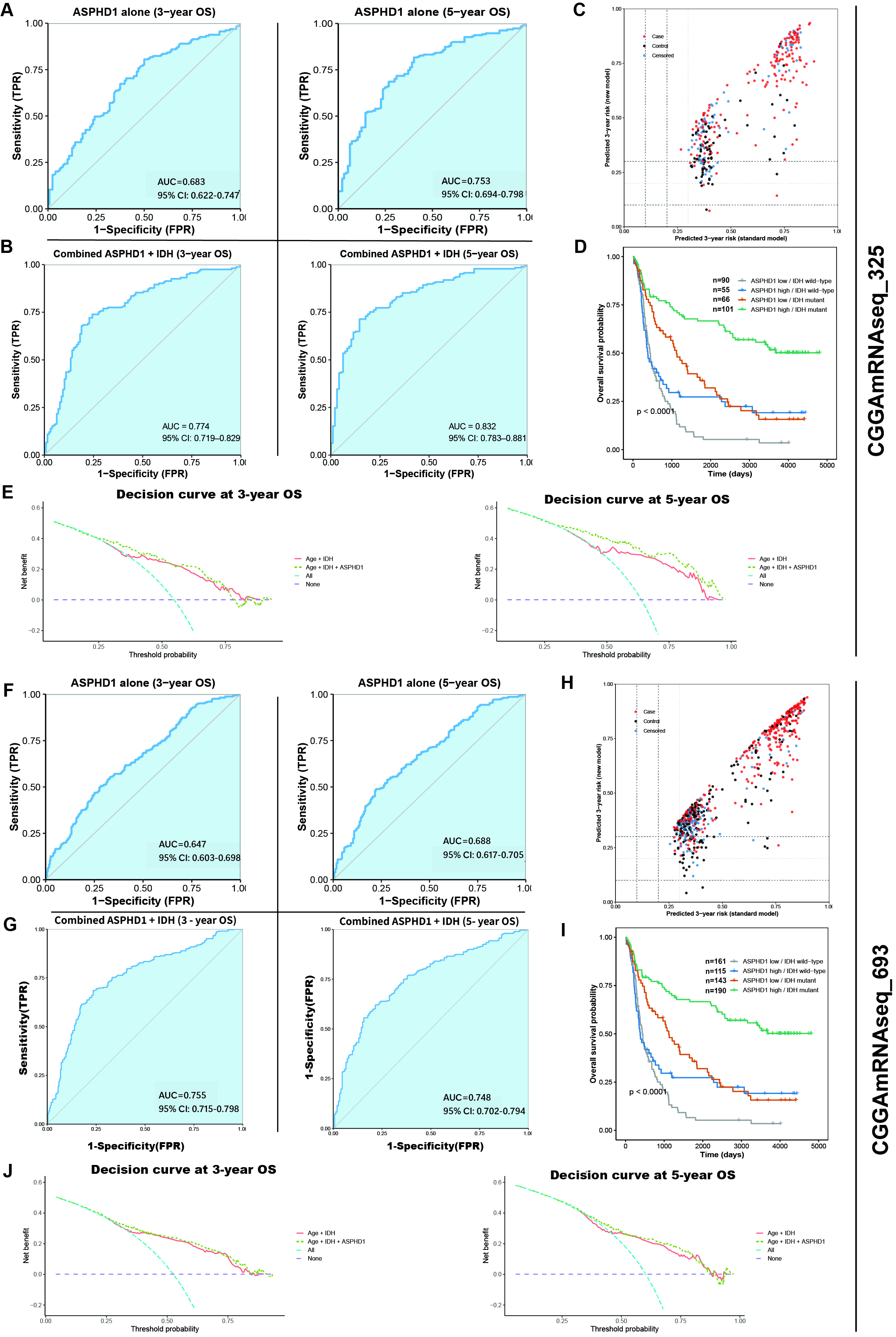

Supplement: Supplementary file 2 [file Image2.tif]

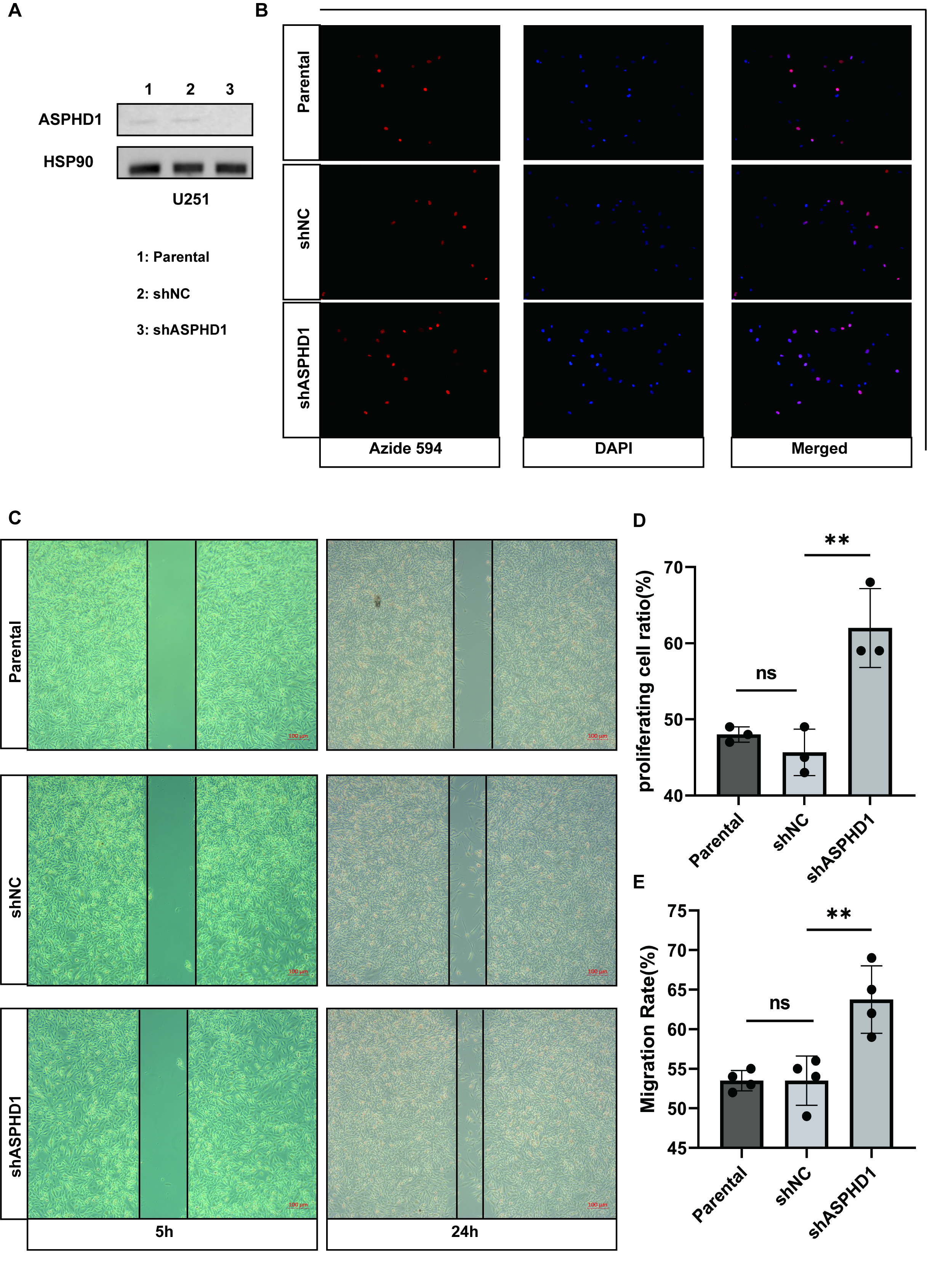

Supplement: Supplementary file 3 [file Image3.tif]

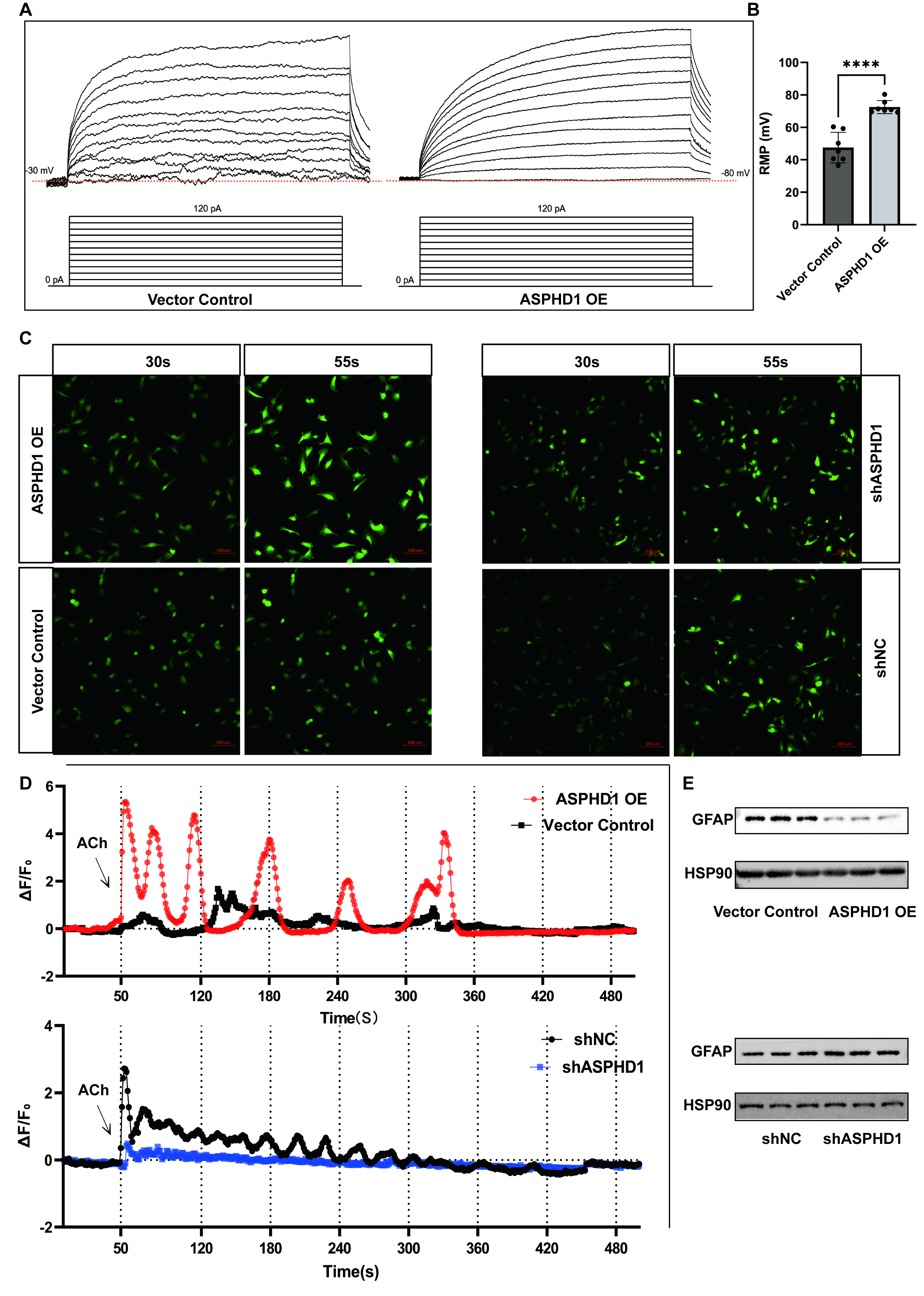

Supplement: Supplementary file 4 [file Image4.tif]
